# Cerebral Activations Related to Audition-Driven Performance Imagery in Professional Musicians

**DOI:** 10.1371/journal.pone.0093681

**Published:** 2014-04-08

**Authors:** Robert Harris, Bauke M. de Jong

**Affiliations:** 1 Department of Neurology, University Medical Center Groningen, University of Groningen, Groningen, The Netherlands; 2 BCN Neuroimaging Center, University of Groningen, Groningen, The Netherlands; 3 Prins Claus Conservatory, Hanze Hogeschool Groningen, Groningen, The Netherlands; University of Montreal, Canada

## Abstract

Functional Magnetic Resonance Imaging (fMRI) was used to study the activation of cerebral motor networks during auditory perception of music in professional keyboard musicians (n = 12). The activation paradigm implied that subjects listened to two-part polyphonic music, while either critically appraising the performance or imagining they were performing themselves. Two-part polyphonic audition and bimanual motor imagery circumvented a hemisphere bias associated with the convention of playing the melody with the right hand. Both tasks activated ventral premotor and auditory cortices, bilaterally, and the right anterior parietal cortex, when contrasted to 12 musically unskilled controls. Although left ventral premotor activation was increased during imagery (compared to judgment), bilateral dorsal premotor and right posterior-superior parietal activations were quite unique to motor imagery. The latter suggests that musicians not only recruited their manual motor repertoire but also performed a spatial transformation from the vertically perceived pitch axis (high and low sound) to the horizontal axis of the keyboard. Imagery-specific activations in controls were seen in left dorsal parietal-premotor and supplementary motor cortices. Although these activations were less strong compared to musicians, this overlapping distribution indicated the recruitment of a general ‘mirror-neuron’ circuitry. These two levels of sensori-motor transformations point towards common principles by which the brain organizes audition-driven music performance and visually guided task performance.

## Introduction

Music is a source of joy for many. In a wider perspective, music, like language, appears to facilitate communication and co-ordination. Humans love to sing *together*, not only in unison but also in harmony. Communal song and dance play an important role in religious and patriotic assemblies, in courtship and parent-child relationships, as well as in war and sport, coordinating affect and affiliation [1], [2]. This specific function of the human brain for music suggests that musical competence is biological, not merely cultural [3]. Next to the manifestation of basic sensori-motor transformations that are so easily recognized in musical behavior such as dancing to the beat, highly sophisticated expression is achieved while playing a music instrument. Also at this high level of expertise, the ability to perform together remains an important characteristic of music behavior [4], [5]. Corporeal synchronization and attuning make it possible to understand another’s intentions and enhance empathic involvement [6], [7]. These interactions between music perception and action illustrate the two levels of general and expert auditory-motor transformation addressed in the present functional Magnetic Resonance Imaging (fMRI) study. The specific aim of our study was to gain insight into the extent to which auditory music perception may activate cerebral regions implicated in expert bimanual keyboard performance.

The strong interrelationship between visual perception and the cerebral organization of motor performance is underscored by the finding that the parietal and premotor cortical regions involved do not maintain strict regional demarcations between perceptual and motor representations [8]–[13]. For example, spatial orientation and direction of movement is processed in joint (dorsal) parietal-premotor circuitry while perceived object shape and prehension is likewise processed in more ventral parietal-promotor regions. These action-associated networks can further be activated in ‘mirror’ fashion as first described in monkey ventral premotor cortex (PMC) [14], [15]. Later, such responses were also observed in more widely distributed parietal-premotor networks of the human brain, evoked not only by action observation [16], but also by aural perception of action sounds such as hammering a nail and sawing wood [17] or the verbal description of action [18]. These stimulus effects are consistent with the notion that the cerebral organization of efficient movements not only employs sensory information by actual feedback but also in an anticipatory mode or by predicted feedback [19]–[24]. The concept of a ‘mirror neuron system’ subsequently lay the ground for models describing the neuronal basis of action recognition and the understanding action of intentions of others in the wider context of social behavior [25], [26] and empathy [27], [28].

In performing on a music instrument, a unique convergence of cerebral functions involving motor preparation, auditory perception, emotional expression and social interaction takes place. It is plausible to assume that the integration of such functions is embedded in neuronal circuitry strongly associated with qualities of a mirror neuron system as described above. During musical performance and perception, interactions of premotor and auditory cortical regions have indeed been proposed to play a crucial role in the integration of feedforward and feedback information [29]. In the last decade, neuroimaging studies have demonstrated that premotor regions of the brain contribute to both perception and production of rhythms and beat [30]–[35]. In this respect, the ventral PMC has been shown to be specifically associated with the perception of musical rhythms during active tapping along with presented stimuli, whereas the mid-PMC and Supplementary Motor Area (SMA) were already activated by unbiased listening [36]. Such ventral PMC responses are consistent with the increased activation during listening to a preferred tempo which was understood to reflect enhanced sensorimotor simulation of the beat frequency, thus facilitating tuning-in to the rhythm of appealing music [37].

To further specify contributions, particularly of parietal and PMC regions crucially implicated in auditory-motor transformations underlying manual music performance, we studied both highly-skilled professional keyboard musicians (further denoted as ‘musicians’) and musically unskilled control subjects (‘controls’) with fMRI. Two-part polyphonic music excerpts were used for auditory stimulation during which subjects had either to imagine playing the excerpts with the corresponding (two) hands on a virtual keyboard (Motor Imagery, MoIm), or to give an ongoing commentary on the presented music (Judgment (Judgm)) without overt vocalization. The advantage of covert motor performance is the absence of actual sensory feedback, thus enabling identification of cerebral activations specifically related to auditory and feedforward somatosensory information implicated in sensori-motor transformations. In contrast to many previous functional imaging studies [38]–[44], our paradigm with strict two-part polyphonic audition and bimanual motor imagery further circumvented a possible bias with covert singing of the leading voice, making it possible to more sharply assess hemisphere-specific contributions to auditory-motor transformations, avoiding possible confounds related to language-associated lateralization in music perception [45]–[47]. Moreover, in the control task, distracting attention from the hands was expected to enhance motor-specific aspects of auditory-motor transformation when contrasted to imagined playing.

When studying auditory-motor interactions in musicians, it should be kept in mind that the use of notation in classical music performance may relatively reduce the direct impact of audition on the motor system. It has been suggested, in this respect, that non score-dependency facilitates melody recognition [48], and that, in particular, improvising musicians recruit motor routines highly dependent on real-time auditory-motor interactions [49]. As we aimed to look for a robust difference between musicians and controls, we selected classically-trained improvising keyboard performers. This resulted predominantly in the recruitment of professional organists.

The hypothesis tested in the present study was that cerebral regions most basically involved in resonating with perceived music, such as the superior temporal cortex and SMA, might be activated in both musicians and controls, while particularly enhanced bilateral activation of the ventral PMC and additional parietal regions was expected in musicians.

## Materials and Methods

### Ethics Statement

The study was approved by the Medical Ethics Committee of the University Medical Center Groningen. All subjects gave written informed consent in accordance with the Declaration of Helsinki (2008) prior to participation.

### Subjects

Twelve professional keyboard musicians and 12 musically unskilled control subjects participated in this study. All 24 subjects were male; in each of the two groups 11 subjects were right-handed. Mean age of the musicians was 43.3y (SD±14.5; distribution 27, 27, 27, 32, 36, 37, 42, 51, 54, 58, 60, 68). The controls had a similar mean age of 43.7y (SD±9.6; distribution 26, 36, 38, 38, 42, 42, 43, 43, 48, 49, 56, 63). Consistent with the inclusion criteria, they were all unable to play any music instrument. None of the 24 subjects suffered from a neurological, ophthalmologic, audiological or upper extremity disorder.

Musicians were professionally improvising, classically trained conservatory graduates (11 organists, 1 pianist) with an average of 25 years of professional experience after earning their Bachelor degree. After finishing their initial music training, they continued their studies, receiving an average of two more degrees in one or more of the following subjects: performance, improvisation, sacred music, composition, theory, music education, and jazz. Seven of the participants were recipients of (on average three) prizes in international organ improvisation competitions. Of those musicians with a teaching practice, three lectured on the faculty of one of the Dutch conservatories. Ten of the eleven organists held positions in a church. All musicians were actively pursuing a performance career.

### Experimental Procedure

The experimental paradigm consisted of performing one of two mental actions while listening to music stimuli. These stimuli were arranged as polyphonic excerpts consisting of two voices of equal rhythmic and melodic salience. Subjects had to either imagine playing the music on a virtual keyboard, without overt movement (Motor Imagery (MoIm)), or give an ongoing commentary on the performance (Judgment (Judgm)) without overt vocalization. The latter was designed to distract attention from the hands, thus enhancing motor-specific aspects of auditory-motor transformation in MoIm, when contrasted to Judgm. Subjects were specifically asked to formulate their commentary verbally, but without actually speaking. They were given complete freedom as to what aspects of the music they would internally ‘talk’ about (see also Text S1) Activations attributed to covert vocalization in Judgm could be expected to be similar in musicians and controls alike.

Half of the 48 music excerpts was completely unfamiliar, having been composed specifically for the experiment by the researchers. Twenty-four ‘familiar’ music excerpts were selected, mainly from the 18^th^ century repertoire (see also Table S1). Two weeks prior to scanning, sheet music of the familiar excerpts was given to musicians to practise, as classical musicians learn their repertoire from sheet music and not from listening to recordings. Controls, who were unable to play a music instrument, received a Compact Disc (CD) recording to achieve familiarity. To ensure familiarity, subjects were instructed either to play through or listen to these pieces daily, keeping track of the number of times they did so. Prior to scanning, subjects were requested to rate the level of acqaintance with the 24 ‘familiar’ pieces on a three-point scale (3 = good, 2 = moderate, 1 = poor). The mean number of times controls had listened to the CD of ‘familiar’ music excerpts in the weeks prior to scanning was 13.6 (SD 7.8) while musicians played through each piece 5.2 times (SD 3.5). The resulting mean familiarity with these stimuli was 2.2 (SD 0.67) in controls and 2.3 (SD 0.83) in musicians. A median of 3 indicated a strong left-skewed distribution in the latter, likely reflecting pre-existing familiarity of musicians with these excerpts.

To avoid activations evoked just by the sound of one’s own instrument, music excerpts were recorded on brass instruments, the bass voice on trombone or euphonium, the treble voice on trumpet or cornet. Students of the Prince Claus Conservatoire recorded these pieces of music in the sound studio of the School of Performing Arts, Hanze University of Applied Science, Groningen. Minor mistakes in interpretation, timing, and intonation were not edited out, allowing room for critical assessment of performance in the second task (Judgm). Recordings were edited to uniform 26s lengths in the studio, including a 2s fade-out and then normalized (max. amplitude −12 dB, Mazzoni normalization using Audacity) and saved in a Waveform audio file format (WAV). Access to the recordings and scores of the unfamiliar excerpts is provided via Sound S1 and Fig. S1. For a baseline condition we used a recording of natural sound (waves of the sea), edited to 14s length including a 2s fade-out and saved as non-normalized WAV audio file. Finally, oral commands were recorded and saved as WAV audio files.

Prior to scanning, an oral instruction on the two tasks MoIm and Judgm was given. During the acquisition of MR images, each music excerpt was presented once, embedded in a 48s cycle containing one of two short (three-syllable) oral commands indicating the task, either MoIm or Judgm, followed by the music excerpt and the baseline sound bite (waves of the sea). The timing was as follows: 2s command, 2s silence, 26s music presentation, 2s silence, 14s baseline sound (waves of the sea) and 2s silence. Four cycles were grouped together in one block, containing all four experimental conditions in random order: (1) MoIm familiar music, (2) MoIm unfamiliar music, (3) Judgm familiar music and (4) Judgm unfamiliar music. In addition, the order of both familiar and unfamiliar musical excerpts was randomized for each subject. Twelve blocks were presented in two runs lasting 20 minutes each, between which a T1 weighted 3D anatomic scan was acquired. A detailed scheme of the scanning protocol is given in Fig. S2.

After the conclusion of the scan, a debriefing was conducted, inquiring into the performance of the tasks. The investigator posed open questions asking for the subjects’ experiences during the conditions of scanning. In addition, subjects were specifically asked whether scanner noise had been excessive. For the latter, the answer was unanimously negative, although two of the musicians mentioned that the acquisition in the middle of the excerpt had distracted them. The time schedule of data acquisition was arranged in such a way that the BOLD responses evoked by the music excerpts were not confounded by the scanner noise (see next section).

### Data Acquisition

Subjects were placed supinely in the bore of a 3T MR system (Philips Intera, Best, Netherlands), which was equipped with an 8-channel phased-array (SENSE) transmit/receive head coil. Lights were turned off and the subject was instructed to keep the eyes closed and not to move during the scan. Hands were positioned on white cushions, visible to the researchers on a television screen, allowing monitoring of undesired hand movements which, however, were not detected during any of the scans.

Sparse sampling of fMRI data started 12s after the onset of each cycle, lasting 2s, and was repeated at regular 16s intervals, meaning that 2s bursts of scanner noise were audible 8s after onset of each music excerpt and again during music fade-out and during fade-out of baseline sound. Subjects listened by means of MR-compatible electrodynamic headhones (MR Confon GmbH, Magdeburg, Germany) [50] that were connected to a standard PC with soundcard. The amplitude of the audio reception was attenuated by 5%. Before each scan, a sound-check was conducted to verify proper volume and stereo presentation by the headphones. Stimuli were delivered using Presentation 14.9.

The functional imaging session was divided in two twenty-minute runs, each consisting of 75 identical high-resolution T2*-sensitive gradient-echo echo-planar imaging (EPI) volume acquisitions (39 slices; repetition time: 16.0s; echo time 30 ms; flip angle 90°; matrix 256×256 in axial orientation; resolution 3.5×3.5×3.5 mm. The acquisition volume was positioned in an oblique axial orientation, tilted backward, parallel to the AC-PC line. The first three scans, prior to the presentation of the stimuli, were only used to achieve stable image contrast and to trigger the start of stimulus delivery. These scans were discarded.

### Data Analysis

Image processing and statistical analysis were conducted with Statistical Parametric Mapping (SPM) [51] version 5 (2005, Wellcome Department of Cognitive Neurology, London, UK; http://www.fil.ion.ucl.ac.uk/spm), running in Matlab (The MathWorks Inc., Natick, MA). The functional imaging volumes were first corrected for motion effects using 3D rigid body transformations. The anatomical images were coregistered to the functional volumes, and all images were normalized into Montreal Neurological Institute stereotaxic space and moderately smoothed using a Gaussian filter of 8 mm full width at half maximum (FWHM).

Cortical activations were rendered onto the surface of a standard MNI brain. For the projection on brain slices, we used the standard MNI brain as well as the mean of the normalized anatomical images obtained from the studied subjects. For the statistical analysis of regional differences in cerebral activation, all conditions were modeled in a blocked design at subject level. To identify the distributions of activations related to cerebral processing beyond primary auditory processing in the conditions 1–4, each of these four conditions was contrasted to baseline interval of natural sound at subject level, after which each contrast was separately analyzed at group level (second level: flexible factorial design; subject, group, condition) using one-sample t-tests. Differences between conditions MoIm (1,2) and Judgm (3,4) within each group, and for each of these conditions between the two groups, were analyzed by making the specific comparisons at second level. The resulting set of voxel values for the indicated contrasts constituted the associated SPM of the t-statistics (SPM<T>). Thresholds were initially set at voxel response height P<0.001 (uncorrected) with extent threshold k = 8 voxels. As within-group comparisons resulted in regional activations that fused into confluent clusters, a FWE-corrected voxel threshold of P<0.05 (k = 8) was applied for these comparisons, demarcating independent clusters of significant activation (P<0.05, volume corrected). For between-group comparisons, clusters resulting from voxel-level analysis at P<0.001 (uncorrected), k = 8, were subsequently assessed for statistical significance after brain volume correction (P<0.05). Conditions were assumed to be dependent and equally variant, whereas subjects were assumed to be independent and equally variant within each or the two groups. In this analysis, differences between familiarity and novelty of music stimuli were not specifically addressed. Plotting the condition effects for regional activations related to MoIm and Judgm, respectively, enabled the assessment of possible interdependency with the level of familiarity or novelty.

## Results

After scanning, the participating subjects were requested to comment on their experiences. Musicians reported continuous bimanual imaging during the Motor Imagery task. Their covert assessment of the performance (Judgment) mostly concerned synchronization, intonation, articulation and style (see also Text S2). Several controls reported difficulty ‘playing’ two parts, focusing only on the melody. One control subject reported imagining playing a violin and one was unable to imagine playing any instrument at all. During Judgment, controls appraised the music in only general terms, e.g. whether they liked the music or what emotion they thought it expressed.

**Figure 1:**
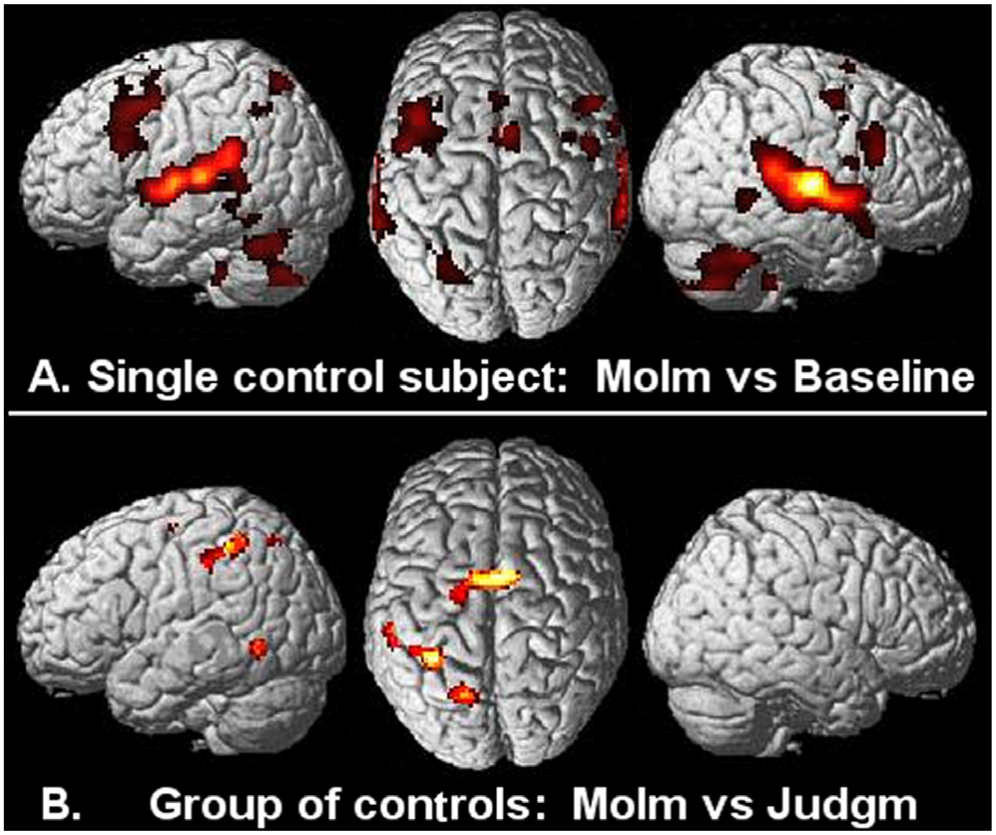
Motor Imagery in musically unskilled controls. A: Increased cerebral activations (SPM<T>) related to ‘Motor Imagery’ of playing perceived music (MoIm), relative to hearing baseline sound (waves of the sea) in a single control subject (P<0.001 voxel-level uncorrected; extent k = 8). Results are rendered onto the surface of a standard anatomical brain volume (Montreal Neurological Institute, SPM 2005). B: MoIm-related increases of activation, compared to listening while covertly commenting on the perceived music (Judgment) in the group of 12 control subjects (P<0.05, cluster-level corrected for the entire brain volume, at voxel-level FWE P<0.05; k = 8). Nomenclature of the activated regions can be inferred from the descriptions in Fig. 2.

Analysis of single subject fMRI data in the musician and control groups showed that bilateral activation of the auditory cortex was the strongest effect of MoIm as well as Judgm (compared to the baseline of natural sound). Moreover, in subjects of both groups, additional PMC activations were generally seen in MoIm as well as in Judgm, regardless of familiarity with the music (Fig. 1a).

**Figure 2:**
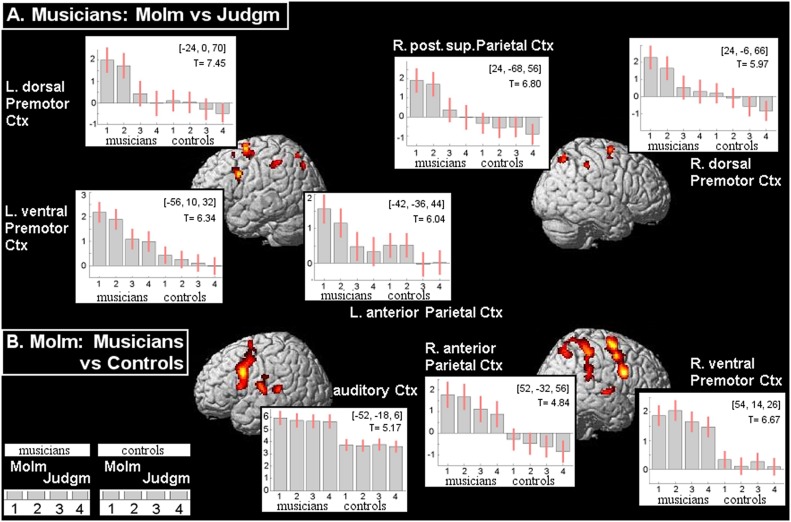
Motor Imagery in professional keyboard musicians. A: MoIm-related increases of regional activation in the group of 12 musicians, when compared to the ‘Judgm’ condition of listening while covertly commenting on the perceived music (P<0.05, cluster-level corrected for the entire brain volume, at voxel-level FWE P<0.05; k = 8). Results are rendered onto the surface of a standard anatomical brain volume (Montreal Neurological Institute, SPM 2005). B: Between-group results showing MoIm-related activation increases in musicians (n = 12), when compared to MoIm in the group of 12 controls (P<0.001, uncorrected; k = 8). Regions with activation increases are labeled by the plots that show the contrast estimates and 90% confidence interval for the effects of interest at the (x,y,z) co-ordinates of maximum condition-related activation and T-value. Effects are provided for musicians and controls during (1) MoIm while perceiving familiar music excerpts, (2) MoIm of unfamiliar music excerpts, (3) Judgm of familiar music excerpts, (4) Judgm of familiar music excerpts. Positive co-ordinate values refer to the distance (in mm) right (x), anterior (y) and superior (z) to the middle of the anterior commissure. L = left, R = right, Ctx = cortex, post. = posterior, sup. = superior.

For the group of musicians, MoIm compared to Judgm was related with a pattern of significant cerebral activations bilaterally distributed over posterior superior parietal and dorsal PMC, together with anterior parietal and left ventral PMC activations (Fig. 2a; Table 1). In controls, this comparison resulted in a pattern of MoIm-related activations that resembled that of musicians only in the left hemisphere, with the exception that only dorsal PMC [x−22, y−10, z 60; T-value 5.61] and no ventral PMC activation was seen (Fig. 1b; Table 2). No significant clusters of activations were found on the lateral surface of the right-hemisphere in controls. On the other hand, activations around the posterior segment of the left inferior temporal sulcus and the supplementary motor area (SMA) were only identified by group analysis in controls and not in musicians (Fig. 1b). The increased activation in the SMA [x−8, y−4, z 60; T-value 6.90] during MoIm in controls, compared to Judgm, was in the same range as the effect size related to both MoIm and Judgm in musicians.

**Table 1:** Cerebral activations in Musicians: Motor Imagery compared to Judgment.

	Left						Right					
	x	y	z	T-value	P(FWE-corr.)	Extent	x	y	z	T-value	P(FWE-corr.)	Extent
Ventral PMC	−56	10	32	6.34	<0.001	148						
Dorsal PMC	−24	0	70	7.45	<0.001	329	24	−6	66	5.97	0.001	131
	−24	14	58	5.47	0.008	13						
Posterior Parietal Cortex	−22	−68	56	6.35	<0.001	47	24	−68	56	6.80	<0.001	131
	−26	−68	30	5.46	<0.008	15						
	−26	−76	44	6.53	<0.001	64						
Anterior Parietal Cortex	−42	−36	44	6.04	0.001	142	46	−36	46	5.54	0.006	56

Co-ordinates refer to the voxel of maximum activation within a significant cluster (P<0.05, FWE cluster-level corrected; extent k 8 voxels). For these maxima, P-values at voxel-level are added. The indicated regions correspond with Fig. 2A. The x,y,z co-ordinates (in mm) are relative to the middle of the anterior commissure. PMC  =  premotor cortex.

**Table 2:** Cerebral activations during Motor Imagery: Musicians compared to Controls.

	Left						Right					
	x	y	z	T-value	P(FWE-corr.)	Extent	x	y	z	T-value	P(FWE-corr.)	Extent
Ventral PMC	−50	8	30	6.05	0.001	759	54	14	26	6.67	<0.001	1213
	*−54*	*4*	*44*	*5.87*	*0.002*		*52*	*4*	*48*	*6.11*	*0.001*	
Dorsal PMC	−26	0	70	4.52	0.187	193	*30*	*−6*	*70*	*5.44*	*0.009*	
Post. Sup. Parietal Cortex							24	−70	54	4.98	0.043	1047
Anterior Parietal Cortex							*52*	*−32*	*56*	*4.84*	*0.068*	
							*44*	*−40*	*62*	*4.69*	*0.110*	
Auditory Cortex	−52	−18	6	5.17	0.022	288						

Conventions are similar to Table 1. Significance level is set at P<0.001 (uncorr.); extent (k) 8 voxels. Resulting clusters that survived cluster-correction for whole brain volume (P<0.05) are reported. Within the confluent premotor cluster, ventral and dorsal clusters can be distinguished. This similarly holds for the left parietal cortex (see corresponding Fig. 2B).

**Figure 3:**
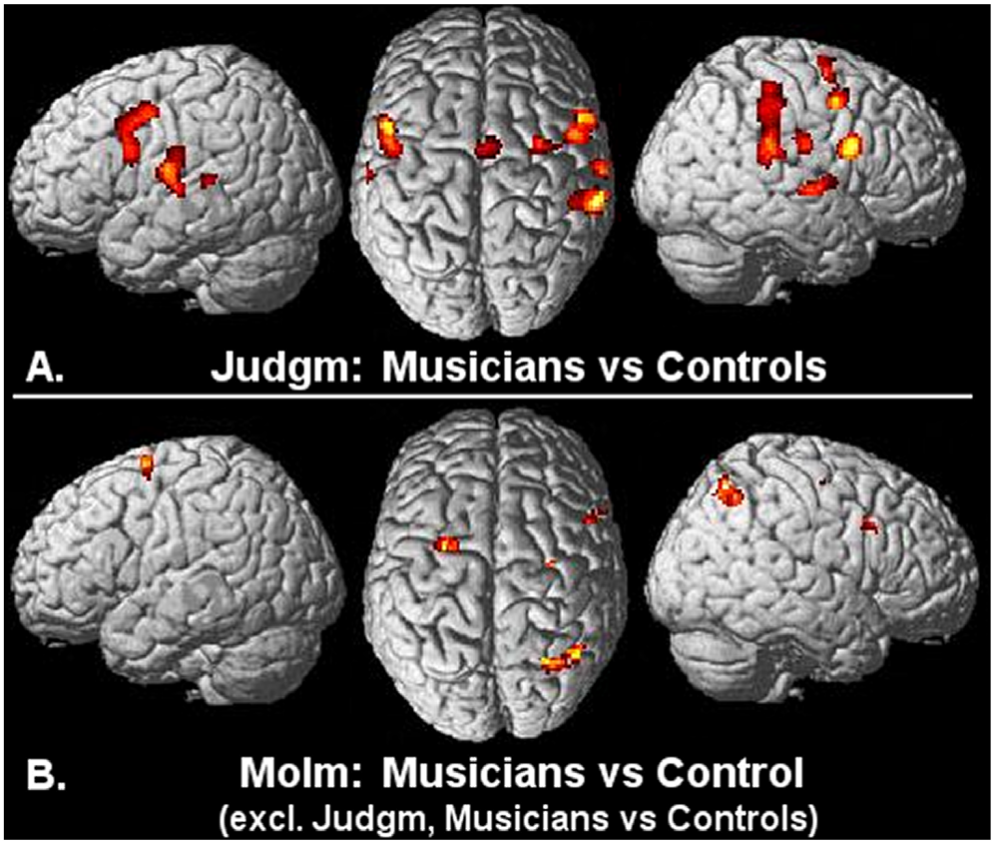
Musicians compared to control subjects. A: Increased Judgm-related activations in musicians (n = 12) compared to Judgm in the group of 12 controls (P<0.001, uncorrected; k = 8). Results are rendered onto the surface of a standard anatomical brain volume (Montreal Neurological Institute, SPM 2005). B: Increased MoIm-related activations in musicians (n = 12) compared to MoIm in the group of 12 controls, with exclusive masking of Judgm-related increases in musicians compared to controls (P<0.001 uncorrected; k = 8). Nomenclature of the activated regions can be inferred from the descriptions in Fig. 2.

Direct comparison between MoIm in musicians and in controls further underscored the unique contribution of the dorsal right-hemisphere parietal–premotor activations to MoIm in musicians (Fig. 2b). The additional activations in the right anterior parietal and ventral PMC of musicians that were identified by this between-group comparison were not entirely MoIm-specific as the two regions showed considerable Judgm-related activations with a magnitude in particularly the right ventral PMC that highly resembled that of MoIm. The direct comparisons of musicians with controls indeed pointed towards similarities between activation patterns in the musicians related to MoIm and Judgm, respectively (Fig. 2b,3a). In addition to the right ventral PMC and the right anterior parietal cortex, this regional overlap in activations particularly concerned the mid-PMC and auditory cortex on the middle portion of the superior temporal cortex, bilaterally. Contrasting MoIm in musicians with the same condition in controls, with exclusive masking for Judgm between the groups, further highlighted the specificity of right posterior superior parietal and bilateral dorsal PMC involvement in MoIm in musicians (Fig. 3b). The profile of condition-related effect sizes in the right posterior superior parietal cortex pointed further towards a unique contribution of particularly this region to MoIm in musicians (Fig. 2a).

The profile of regional effect sizes demonstrated that the basic activation pattern related to MoIm in musicians was hardly influenced by familiarity or novelty of the presented music excerpts (Fig. 2). Such plots further illustrated that for both musicians and controls, anterior parietal activations in the left hemisphere were increased in MoIm relative to Judgm. Right anterior parietal activations, with highly similar magnitudes for MoIm and Judgm in musicians, did not occur in controls (Fig. 2b).

Contrasting Judgm to MoIm did not result in significant increase of activation, neither in musicians, nor in controls. In musicians, this comparison resulted in only a regional increase of activation located at the anterior portion of the left superior frontal gyrus (x−14, y 56, z 30; p<0.001, uncorrected).

## Discussion

The two groups studied in the present experiment differed mainly in their ability or inability to play a music instrument. While control subjects are completely unable to play a music instrument, musicians had years of experience and training doing so. We demonstrated that control subjects recruited dorsal parietal-premotor regions implicated in motor control, including the SMA, while imagining playing the music they heard (on an instrument they were unable to play). The distribution of this MoIm-specific cortical activations was fully lateralized to the left hemisphere, when contrasted to Judgm, and did not include the ventral PMC. MoIm in professional musicians revealed additional left-sided activations in the ventral PMC and anteriorly in the inferior parietal cortex, together with right dorsal parietal-premotor activations. This differential parietal-premotor involvement in the two groups illustrates that the cerebral motor system can indeed be rather easily facilitated by listening to music, consistent with the concept of a ‘mirror-neuron system’ [25], [52], while the specification of distinct movements requires expert-unique computations in additional parietal-premotor regions. These regions may thus be seen as an interface serving the interactions between representations of embedded musical skill and auditory stimuli. Moreover, similarity between the magnitudes of the MoIm and Judgm activations, particularly observed in musicians’ right ventral premotor cortex suggests that expert music-perceptual analysis is intrinsically associated with covert music performance.

### Expertise in the Auditory Cortex of Musicians

Activation of the auditory cortex in the two groups underscored the fact that music evoked stronger responses than the base-line ‘sounds of the sea’. This can be logically explained by the more complex frequency composition of music [53]. When balanced for acoustic features, music stimuli nevertheless evoked stronger activation in the middle segment of the auditory cortex in musicians than in the auditory cortex in controls. This location was virtually identical to the music-specific region described by Angulo-Perkins et al. [54], just posterior to the representation of human sound in their study, which has particularly been implicated in pitch height processing [55]. The fact that the auditory cortex effect was task-independent, i.e., responses to MoIm and Judgm were similar and without a familiarity effect, may support a mechanism of early-stage over-specialization for musical sound in musicians [53], [56], unrelated to possible top-down processing [57]. Such regional specialization, irrespective of possible top-down effects, is consistent with expert-related segregation between representations of sound in music and speech, respectively [58].

### Mirror-neuron Circuitry

Mirror-neurons in circuitry underlying auditory-motor transformation involved in oral action have been proposed to play a role in the evolution of human speech [52]. This may similarly hold for the evolution of human capacities for music and dance which, just as for speech, have failed to evolve in other primates [59]. This biologic predisposition in humans [3] is characterized by entrainment to beat as well as to melodic contour [60]. The MoIm-specific activations in controls may thus reflect the neuronal underpinning of perceiving music as an affordance, i.e. as something dance-able, clap-able, sing-able, whistle-able or hum-able [32], [61]. Given the prominent SMA activation within this pattern, MoIm-related activations in controls may well represent action-mediated perception of beat [30], [34].

Musicians were expected to perceive music not only as clap-able or sing-able but also as ‘play-able’. Support for a specific neuronal underpinning of such musical skill can be obtained from the previously demonstrated pattern of activations in musicians during imagined playing overlearned music, comprising SMA and bilateral parietal-premotor regions [62]. However, that pattern may have included activations related to more general imagery of hand movement which is known to recruit similar bilateral circuitry [63]. On the other hand, it has been demonstrated that passive listening to music evoked auditory-parietal-premotor activations when subjects had attentively listened to this music before, while the premotor activation even further increased when such music pieces had actually been practised in the week preceding scanning [64]. This supported the concept that a similar mode of mirror-neuron processing is implicated in object-action and sound-action transformations [64], while such sound-action conversion may be enhanced by training [64], [65], [66]. The present study corroborates and extends previous results, particularly as specificity of the activations related to MoIm in musicians was achieved by comparisons both with Judgm and between the two groups.

### Vertical Pitch to Horizontal Keyboard Rotation in Musicians

The right posterior superior parietal cortex was the unique location in which activation only increased during MoIm in musicians, without an effect of Judgm. In the following text we will motivate our view that this activation represents a kind of mental rotation of heard sounds, used by the musicians to play them at the keyboard. The right-sided parietal effect in the bimanual task is not explained by isolated left hand performance. Neither is it reasonable to claim that the left hand polyphonic parts were more demanding or musically more important that the right. Its posterior location points at a higher-order contribution to motor control [11], [22], [67]–[69] while right-sided lateralization provides an argument for the involvement of spatial transformation [13], [70]–[72]. As the ‘spatial’ dimension of pitch in music has been shown to be perceived as vertical [73] and the imagined hand movements on the virtual keyboard are along the horizontal axis, this implies that the pitch-to-performance transformation would involve a mental rotation [74]. This agrees with ideas concerning a general code of spatio-temporal processing implicated in the cerebral embedding of music [75], [76], and the role of the parietal cortex in musicians, in achieving linear ‘spatial’ operations when transposing a melody to a different key [77]. The ability to recruit such parietal function in order to achieve the audition-based virtual motor task thus appears to be a highly specific ability of musicians. Incorporation of right posterior parietal information in a wider parietal-premotor network is logically mediated by the strongly interconnected dorsal PMC in the same hemisphere [11], [78], [79]. Coherence of these MoIm activations in musicians was particularly well demonstrated by the comparison with MoIm in controls while excluding Judgm-related increases in musicians compared to controls, which revealed a specific pattern comprising the right posterior parietal cortex and dorsal PMC, bilaterally.

### Ventral PMC Function

In control subjects, the left dorsal PMC also showed increased activation during MoIm (compared to Judgm), but this increase was much weaker than in musicians. As described above, the effect in controls was inferred to reflect potential recruitment of nonspecific motor responses. In contrast, in the ventral PMC of this hemisphere, MoIm compared to Judgm only evoked an activation increase in musicians and not in controls. This may well reflect the ability of musicians to organize more specific movements given the functional involvement of the ventral PMC with prehension and selection of distal upper limb movement [12]. The ventral PMC activation may thus represent a general mechanism by which the experts master the code of expressing music in distinct finger movements. Left hemisphere dominance, in this respect, seems consistent with the left-hemisphere dominance in skilled movement.

One might argue that the left ventral PMC activation reflects dominance of the melody that was imaginarily played by the right hand [80]. This suggestion is refuted by the similarly strong MoIm-related activation in the musicians’ right ventral PMC. In the latter, however, Judgm-related activation equalled the MoIm effect, while Judgm evoked less strong activation in the left ventral PMC. The activation increases related to Judgm in musicians, compared to controls, were distributed over the ventral PMC, the antero-inferior parietal cortex and the auditory cortex in both hemispheres. Such a perisylvian pattern is consistent with the literature on equivalent analyses of syntax structure in melodic contour and language [30], [81]–[84]. On the other hand, in contrast to lateralized language functions, hemisphere specialization related to music analysis cannot be unequivocally concluded from the literature. Comparing professional musicians and actors has even demonstrated that perisylvian brain regions implicated in speech may gain a music-specific function depending on long-term auditory-motor expertise [58]. In our study, the strongest Judgm effects were in the right perisylvian regions of musicians, with effect sizes close to those of the MoIm activations. One may speculate whether this right-lateralized perisylvian similarity of MoIm and Judgm activations reflects global harmonic processing beyond melodic contour [85]–[87]. No activations were significantly stronger in Judgm than in MoIm, which provides an argument supporting the idea that expert music-perception analysis rather automatically induces elements of covert music performance. Considering such a motor component, the similarity between the musicians’ response profiles in the ventral PMC and antero-inferior parietal cortex of each hemisphere is constistent with ventral parietal-premotor interconnections associated with goal-directed hand movement [9], [88]. In the present task conditions, activation of the antero-inferior parietal cortex in musicians is best explained by its involvement in neuronal processing of the predicted sensory consequences of movement [21], [24], thus serving to prepare the appropriate finger movements on the keyboard.

### Lateralization in ‘Bimanual Performance’

Our paradigm was designed with strict two-part polyphonic audition and bimanual motor imagery. Such ‘double-task’ characteristics thus avoided e.g. a left-hemisphere bias due to right-hand performance only [38] or covert singing of either a single melodic line or the dominant melody in a homophonic composition [44]. The observed lateralized activations, particularly in the right parietal cortex related to MoIm, were therefore attributed to higher-order components in the organization underlying manual music performance. In this respect, our findings add to, rather than contradict, previous studies emphasizing left-hemisphere dominance in musical expertise. Still, a discrepancy may seem to exist with the fMRI study of Itoh et al. [89] in which a bimanual paradigm revealed particularly left parietal cortex activation. They suggested that left-lateralization might be attributed to the fact that subjects were reading from the score. In addition, a silent piano keyboard was used which might imply performance with only somatosensory feedback and without actual audition and music production. This may have led to particularly left-lateralized activation representing the dominance of executing general skill, overruling the sensori-motor transformations underlying the auditorily elicited manual expression of music.

## Conclusion

Keyboard performers who master the skill of playing aurally perceived music appear to recruit an acquired instrument-related motor repertoire from circuitry particularly embedded in parietal-premotor cortical regions additional to a more general ‘mirror-neuron’ circuitry. The latter is also elicited in musically unskilled subjects, although less robustly. Unique for musicians was the finding that the perception of music with the intent of playing involves a spatial transformation from vertical pitch space to horizontal keyboard space, associated with right postero-superior parietal activation. In this respect, general rules of spatial transformation in higher order motor control appear to serve aurally elicited manual music performance. The combination of such spatial processing with auditory-motor transformations that occur in a simpler ‘mirror’ fashion indicates that similar principles of neuronal processing underlie instrumental music performance by ear and visually guided task performance.

## Supporting Information

Figure S1 Sores of the new composed (unfamiliar) music excerpts.
(DOC)

Figure S2 Scheme of scanning a single trial.
(DOC)

Table S1 Titles of familiar music excerpts.
(DOC)

Text S1 Task instructions.
(DOC)

Text S2 Debriefing after scanning.
(DOC)

Sound S1 Recordings of the new composed music.
(ZIP)
