# Supplementary figures and images for: Cerebral Activations Related to Audition-Driven Performance Imagery in Professional Musicians
Source: PLoS One. 2014 Apr 8;9(4):e93681. doi: 10.1371/journal.pone.0093681 (PMC3979724; doi:10.1371/journal.pone.0093681)

**Figure S1. SORES OF THE NEW COMPOSED (UNFAMILIAR) MUSIC EXCERPTS.**


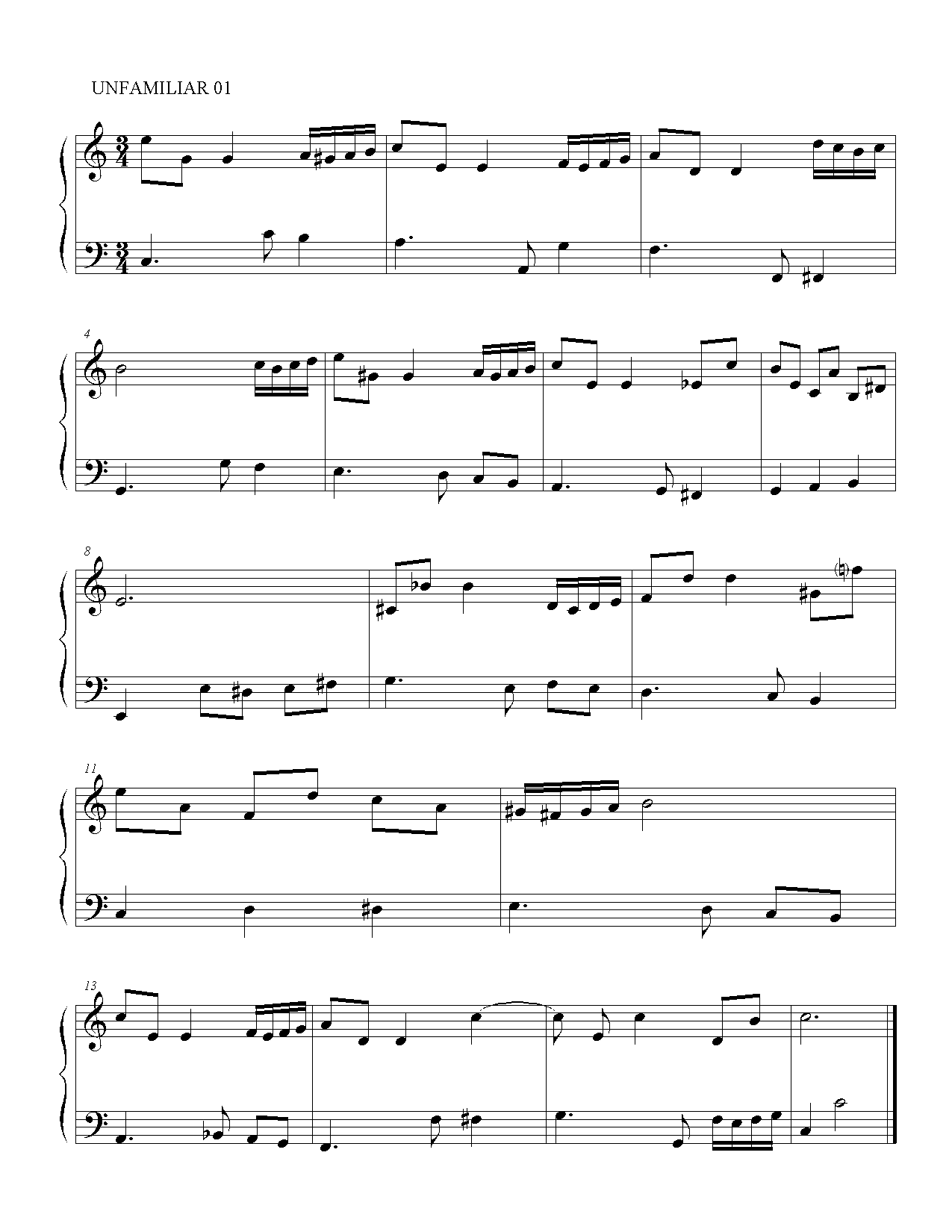

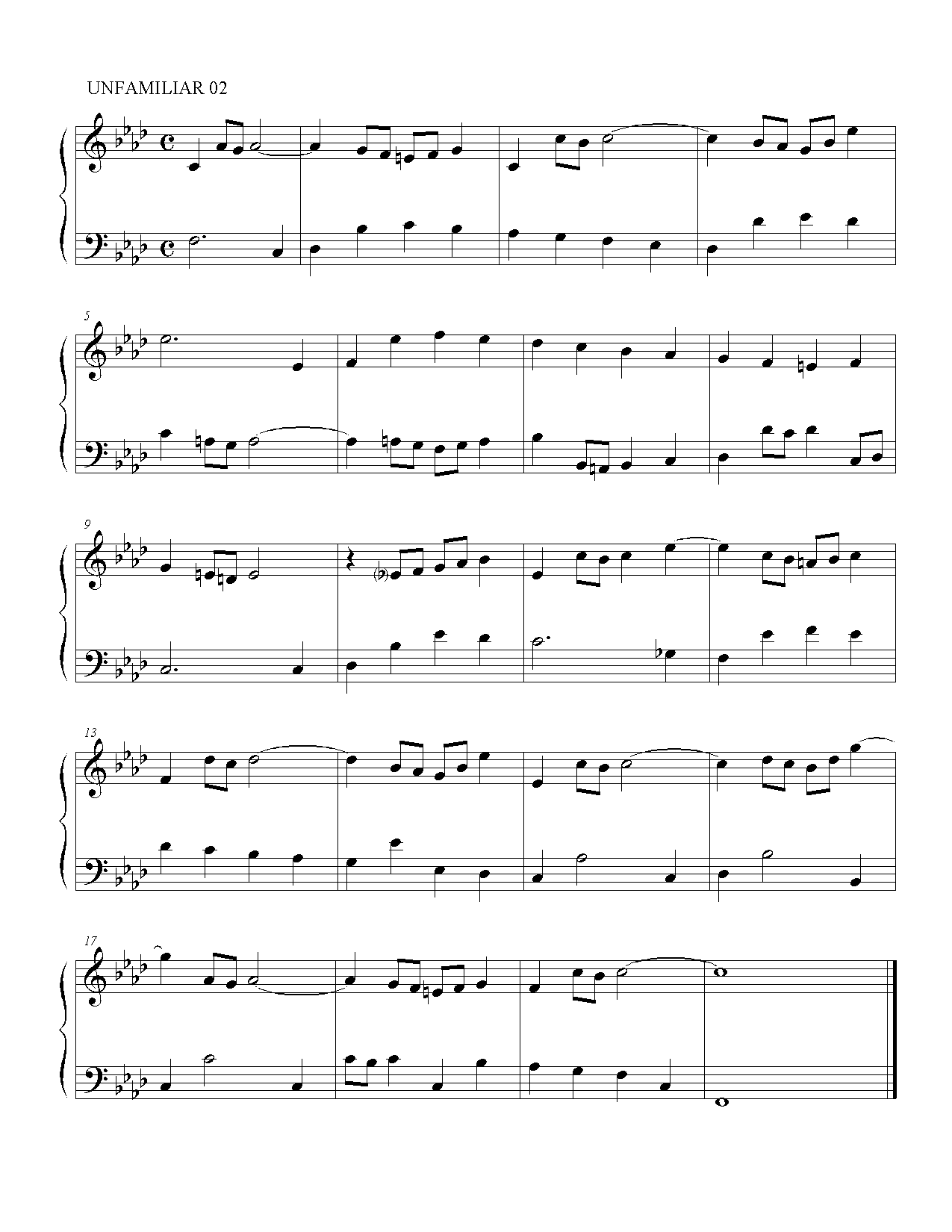

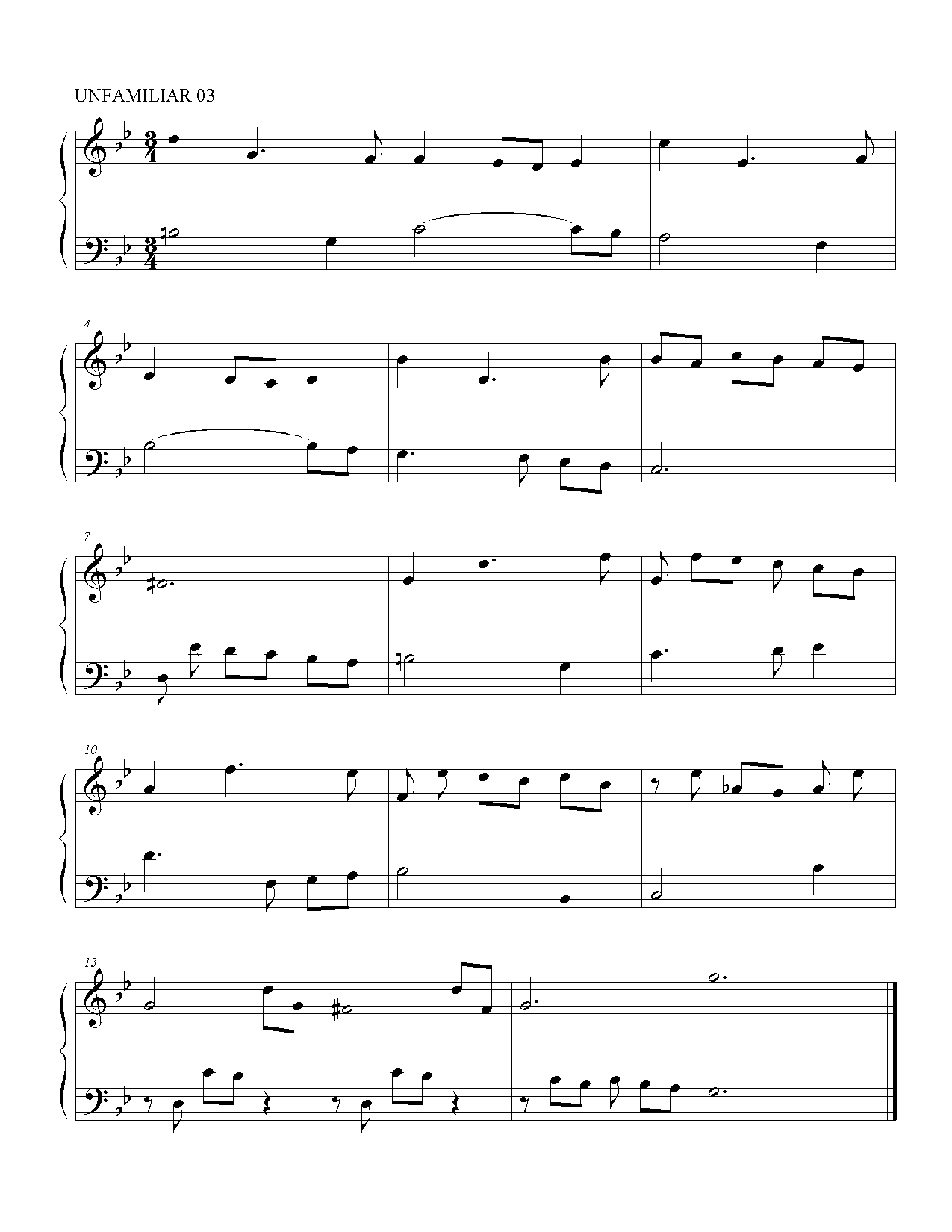

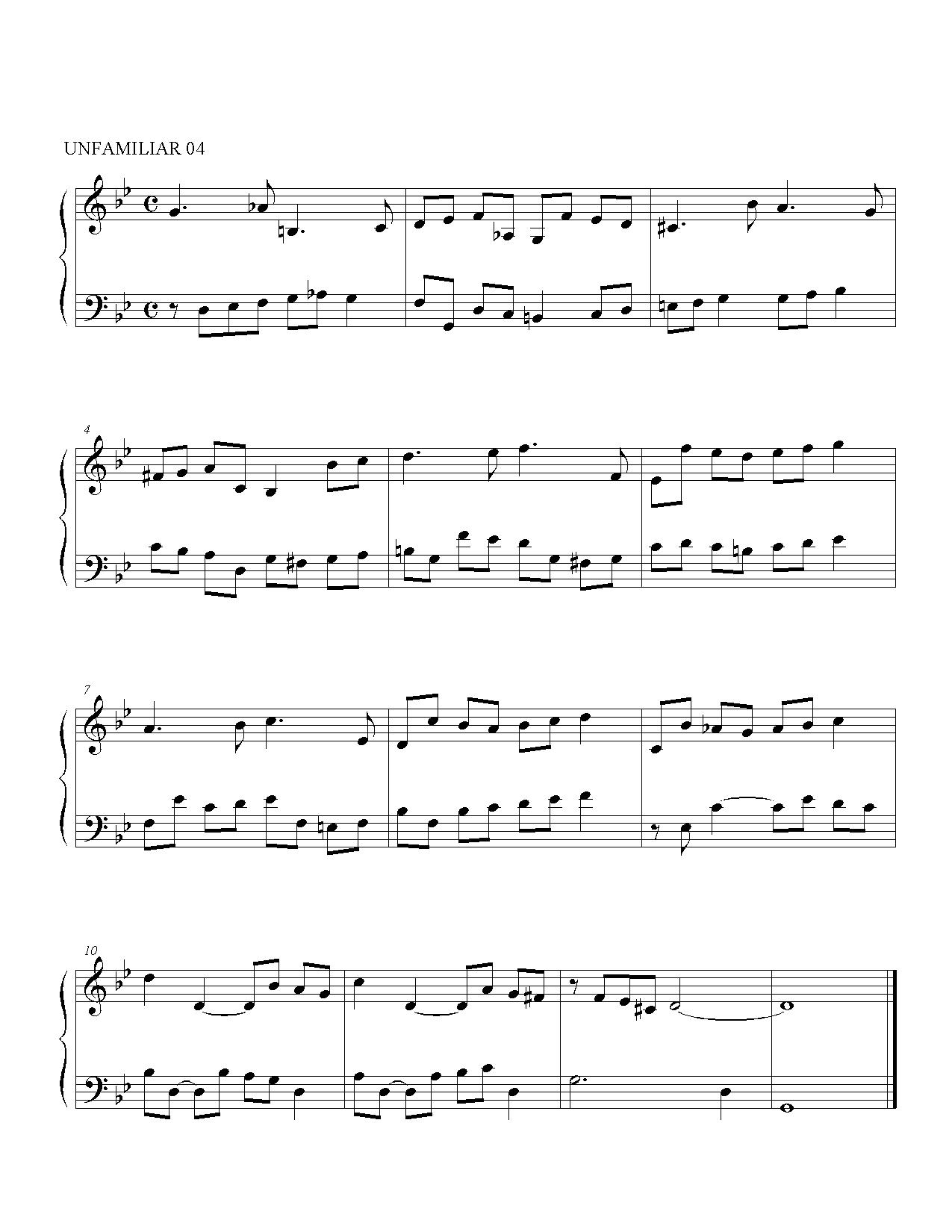

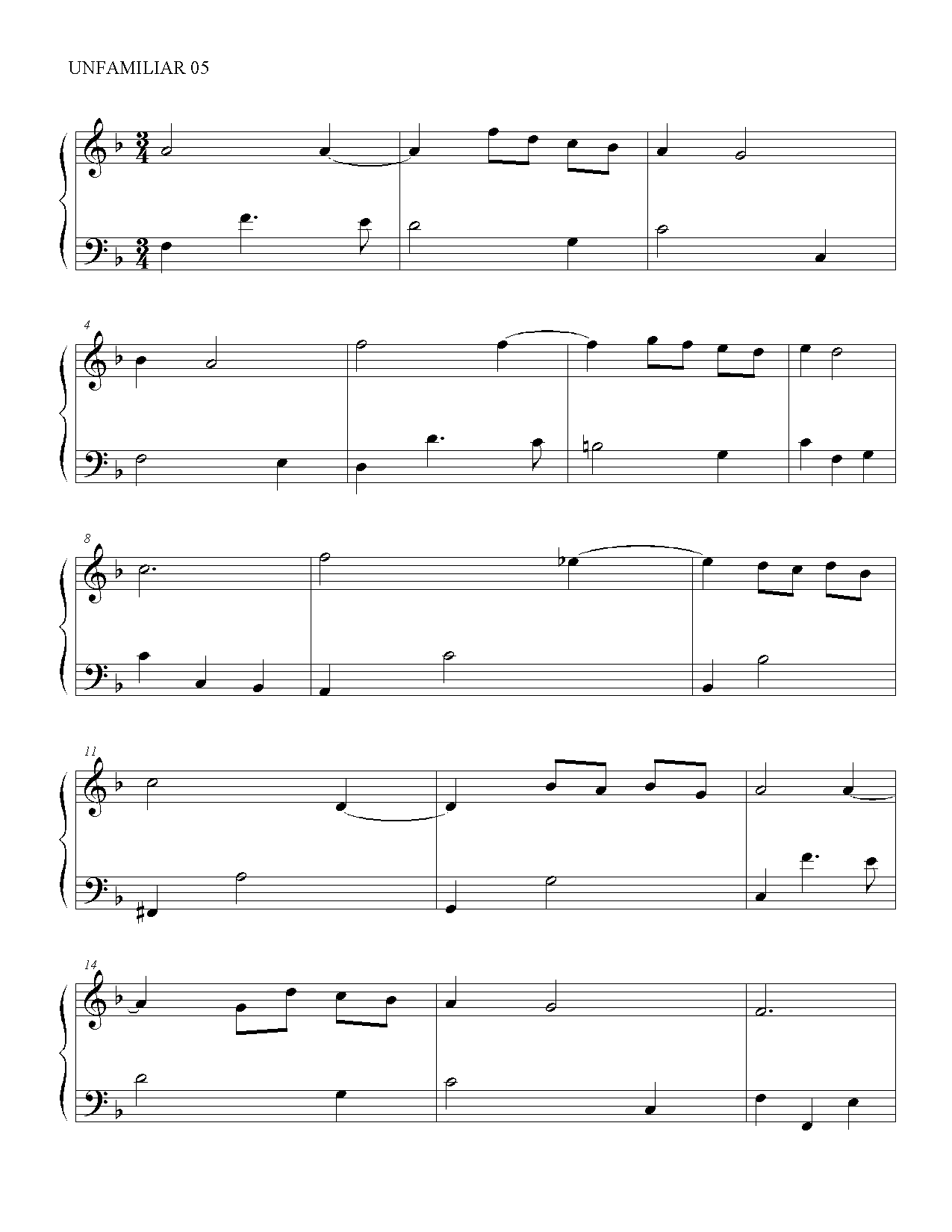

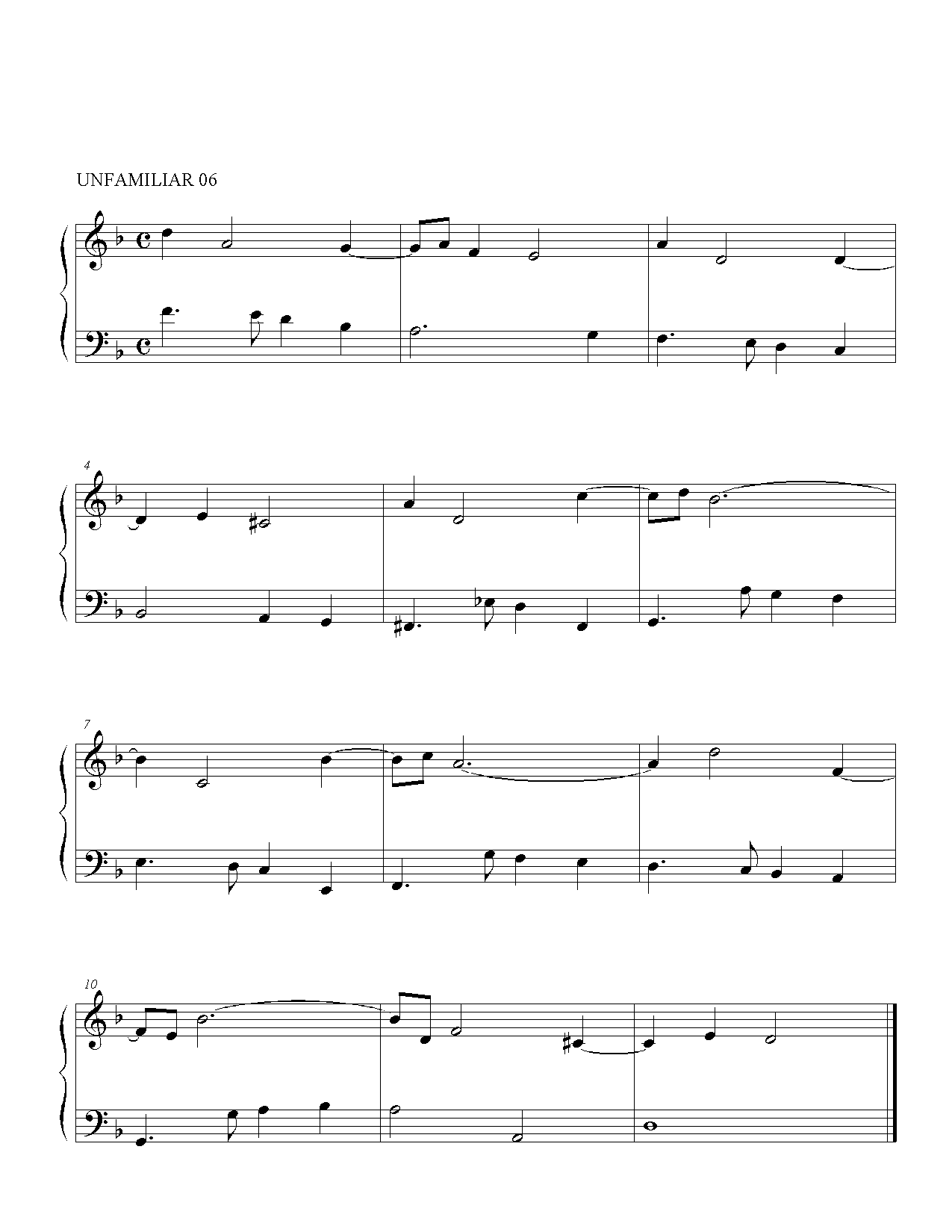

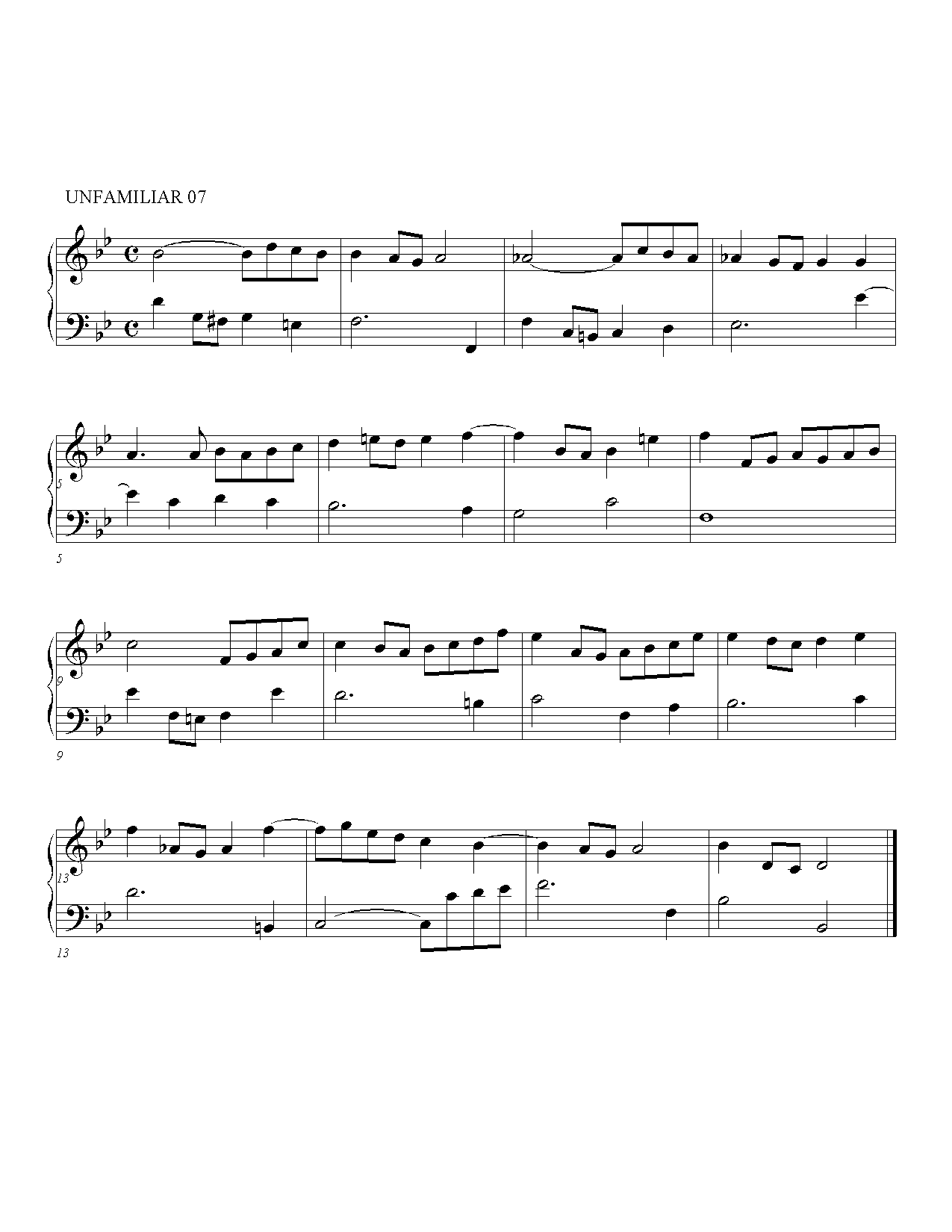

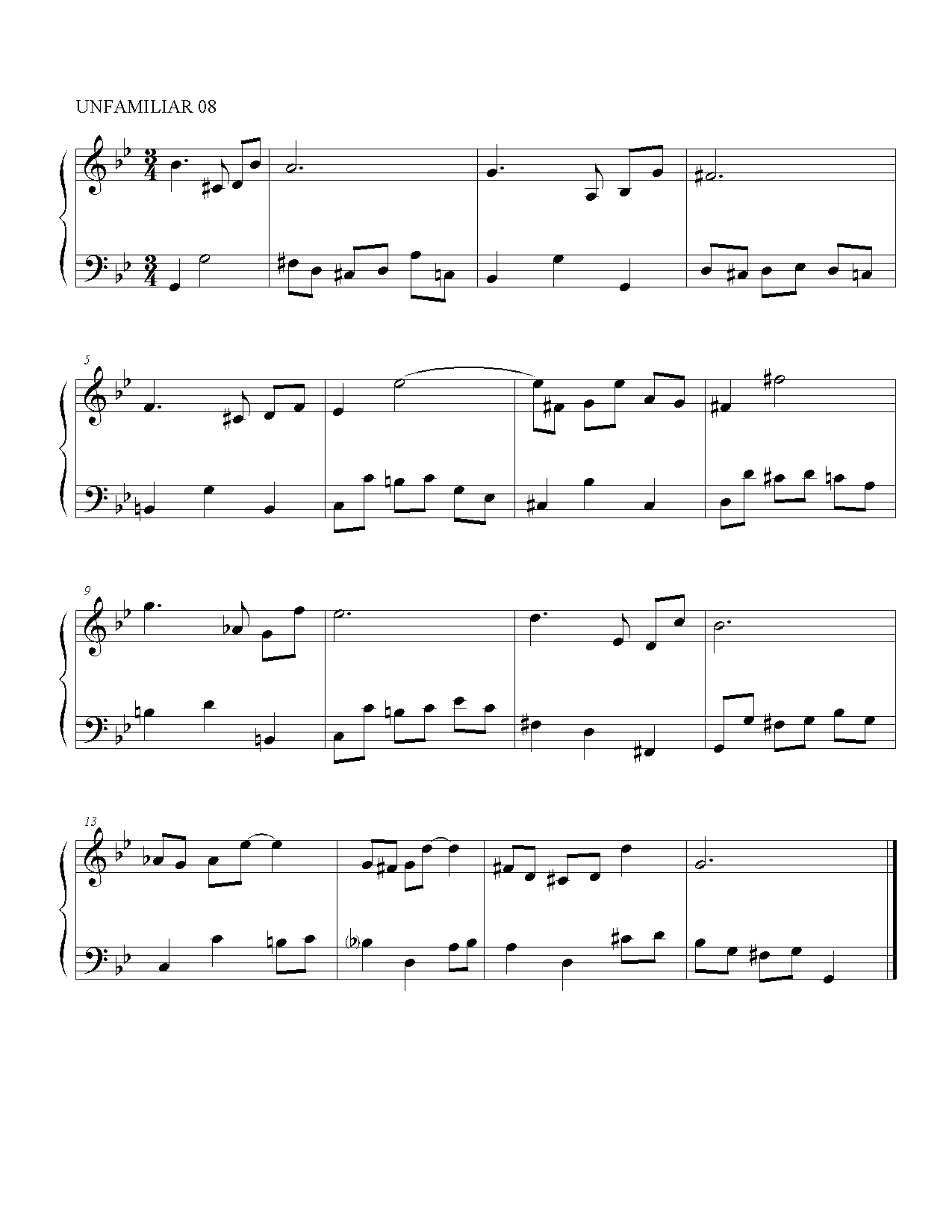

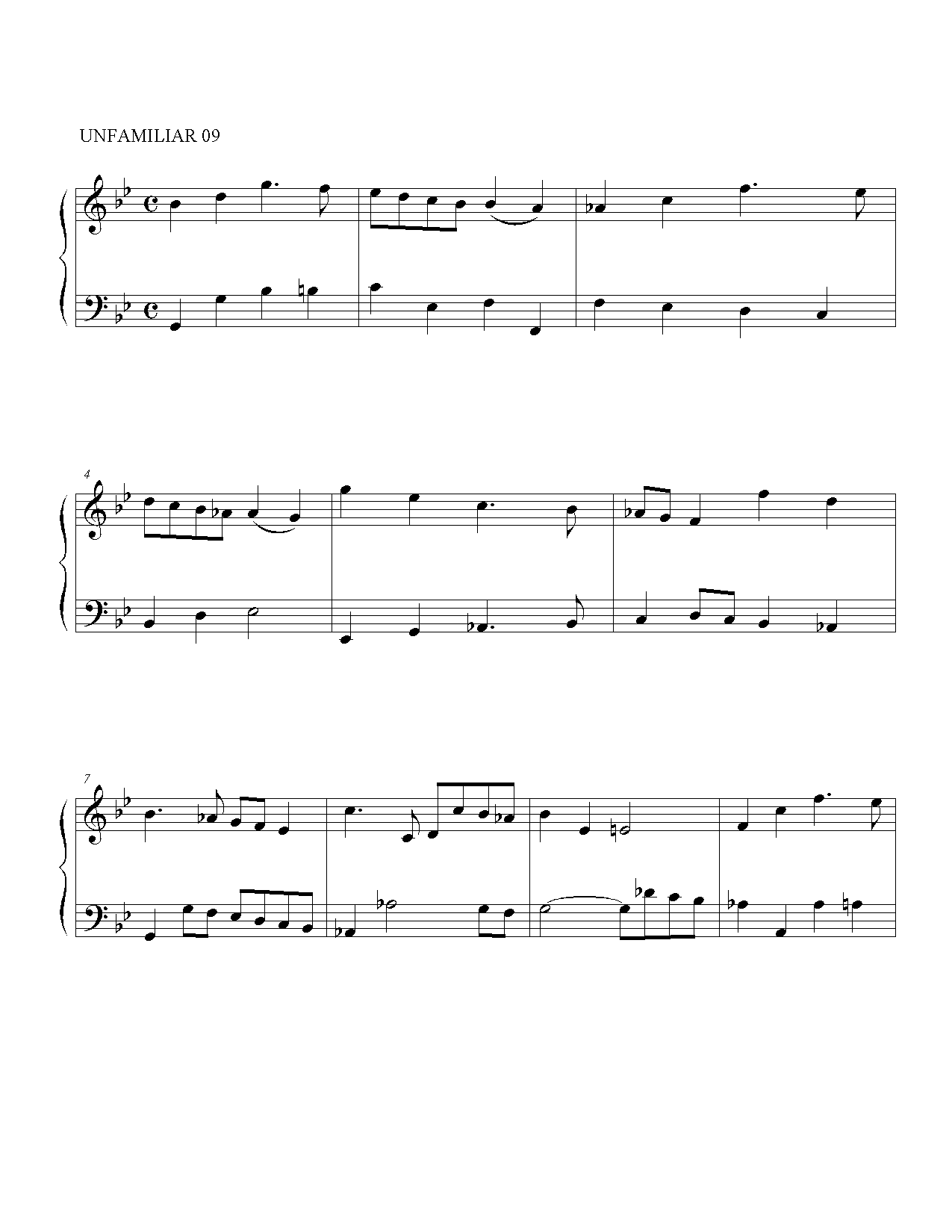

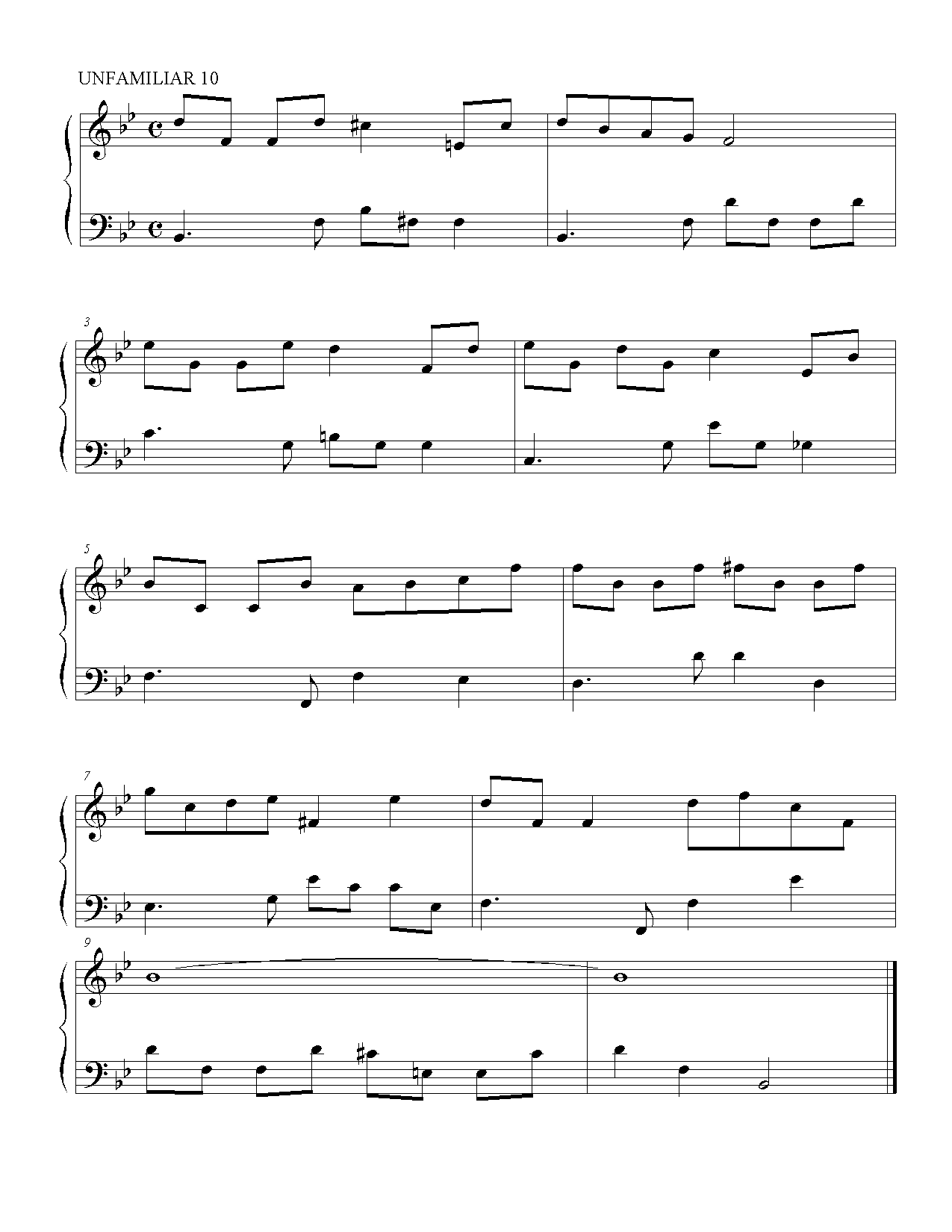

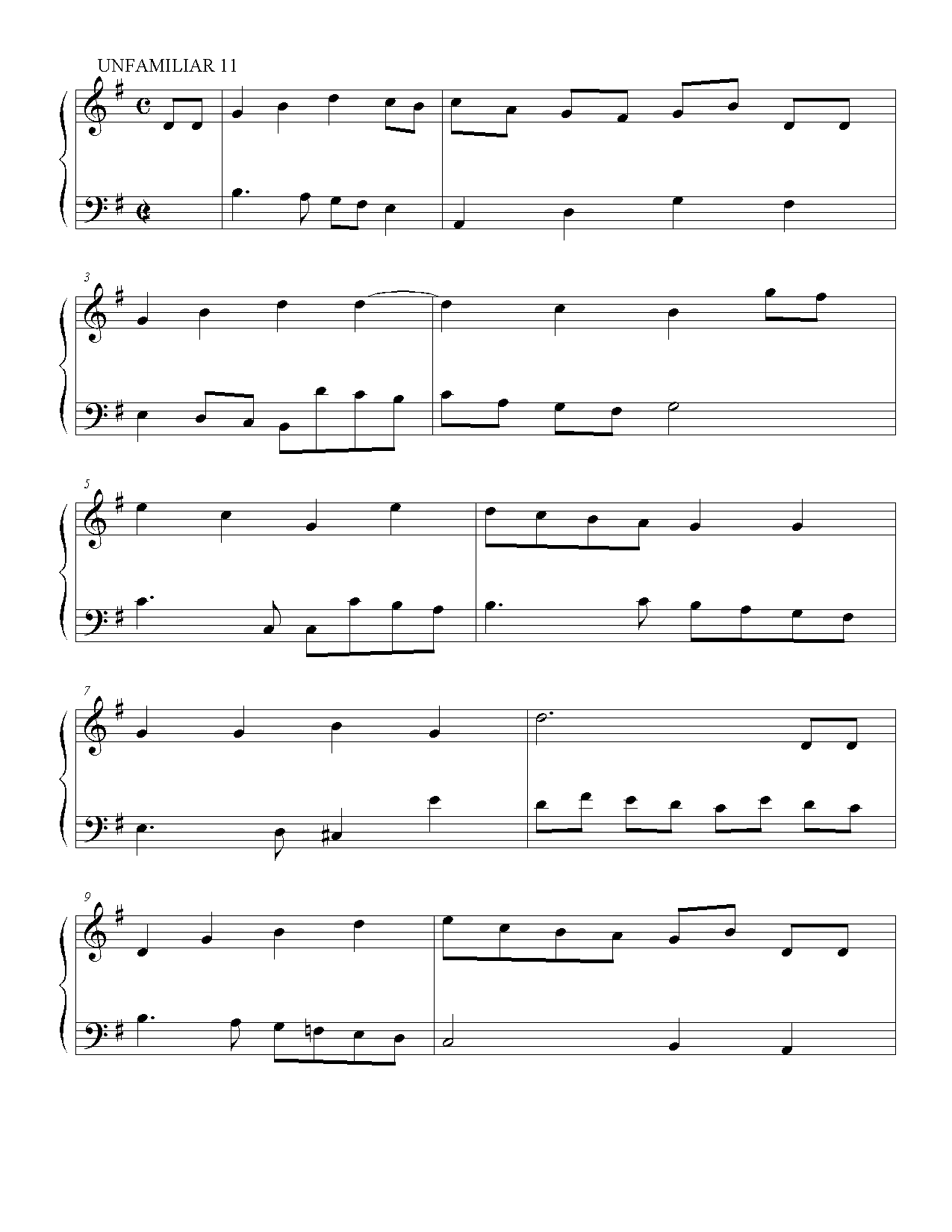

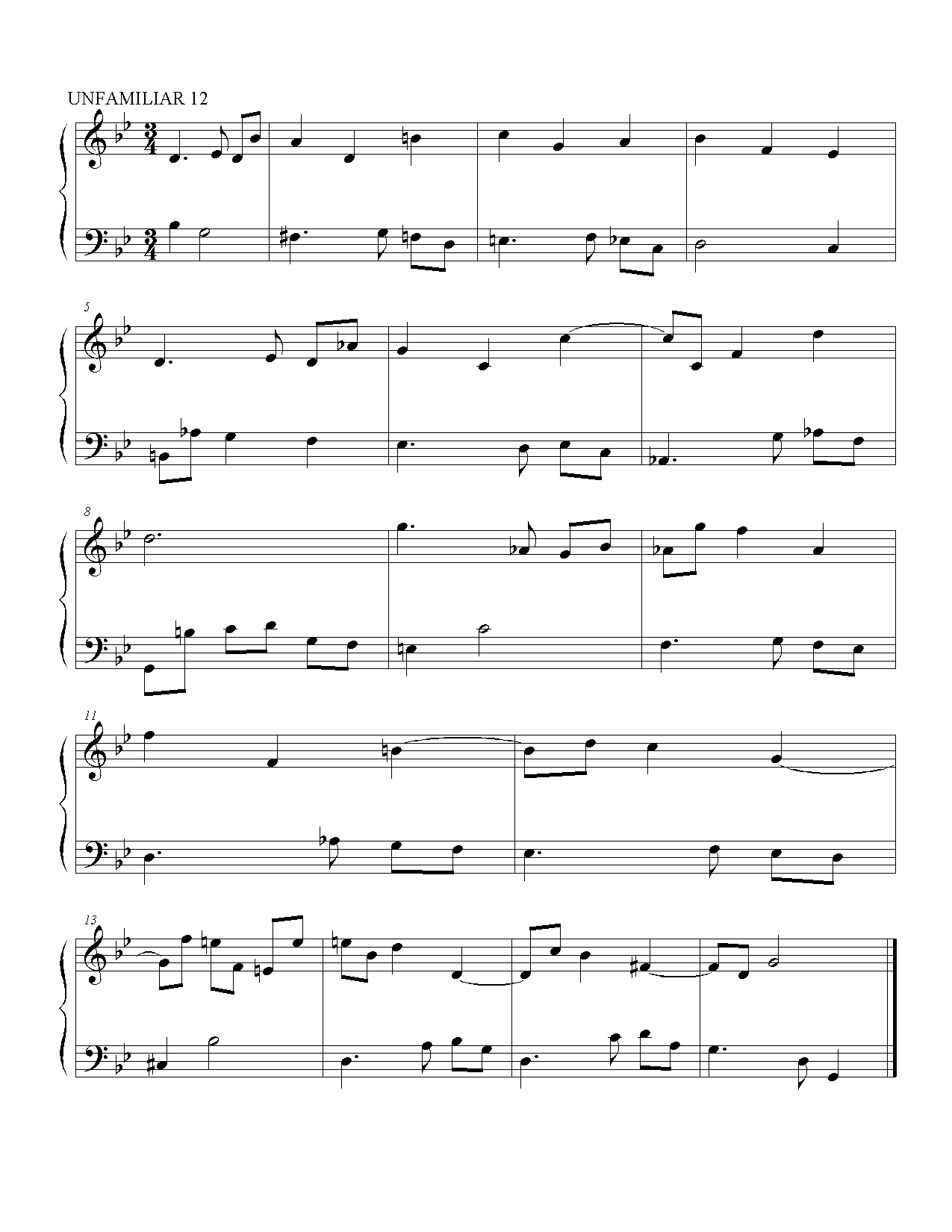

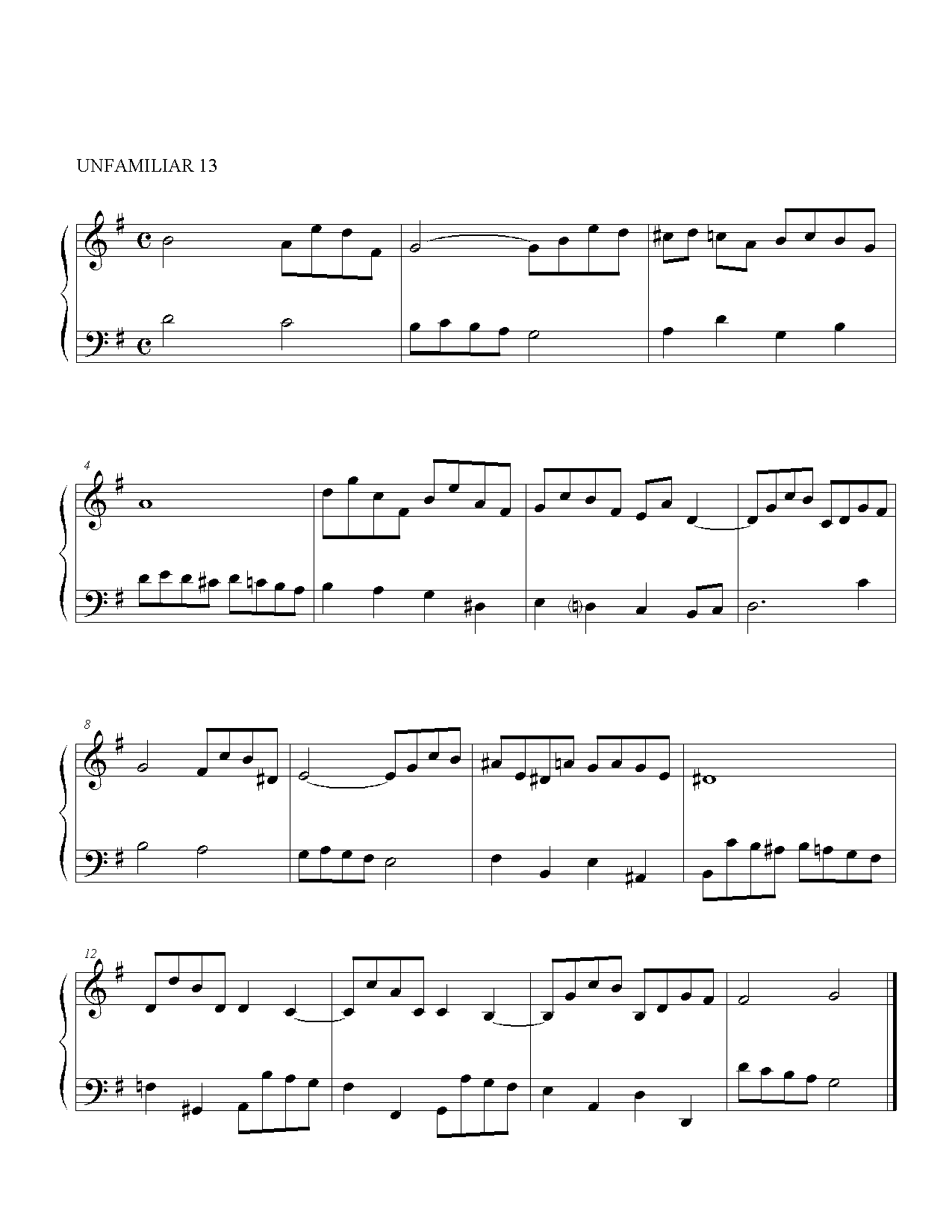

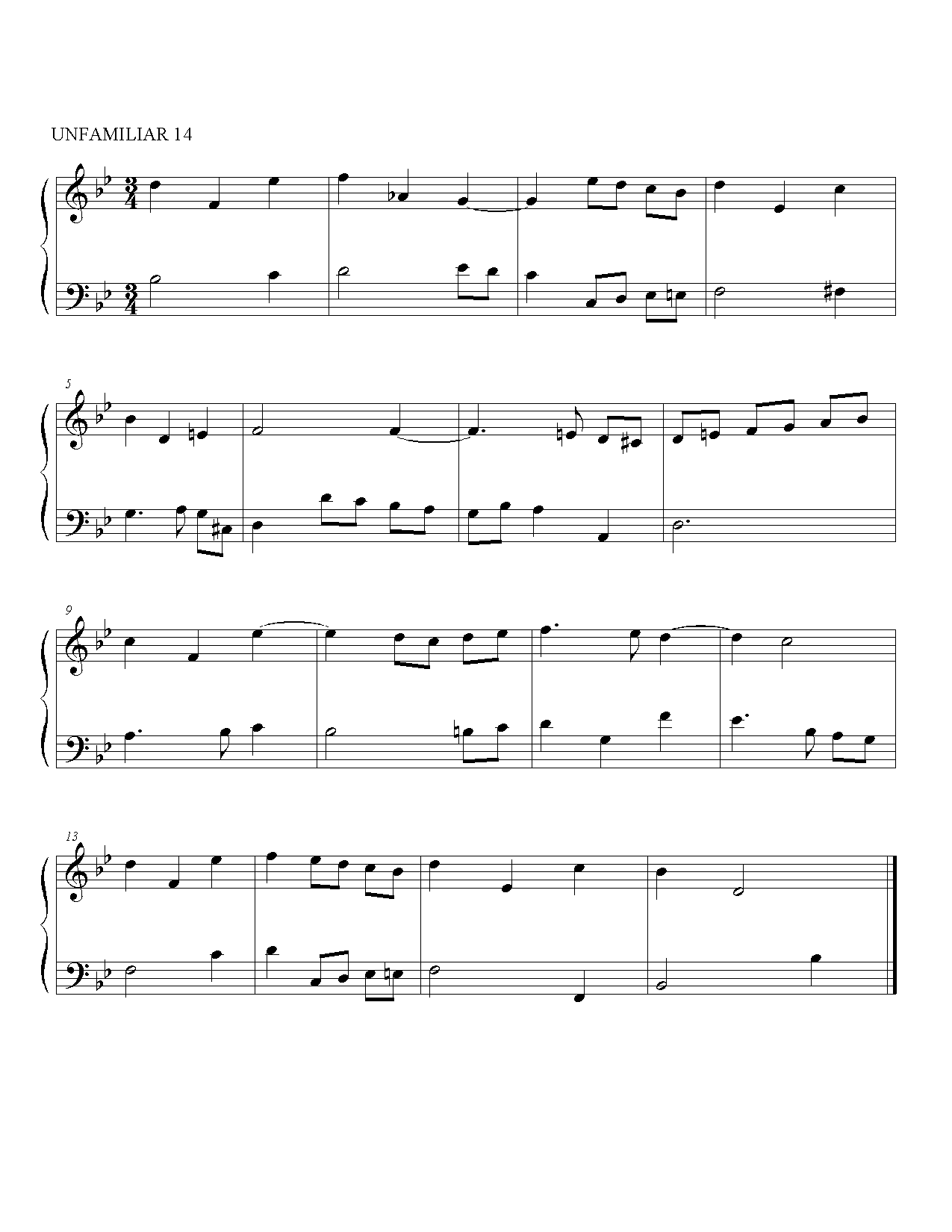

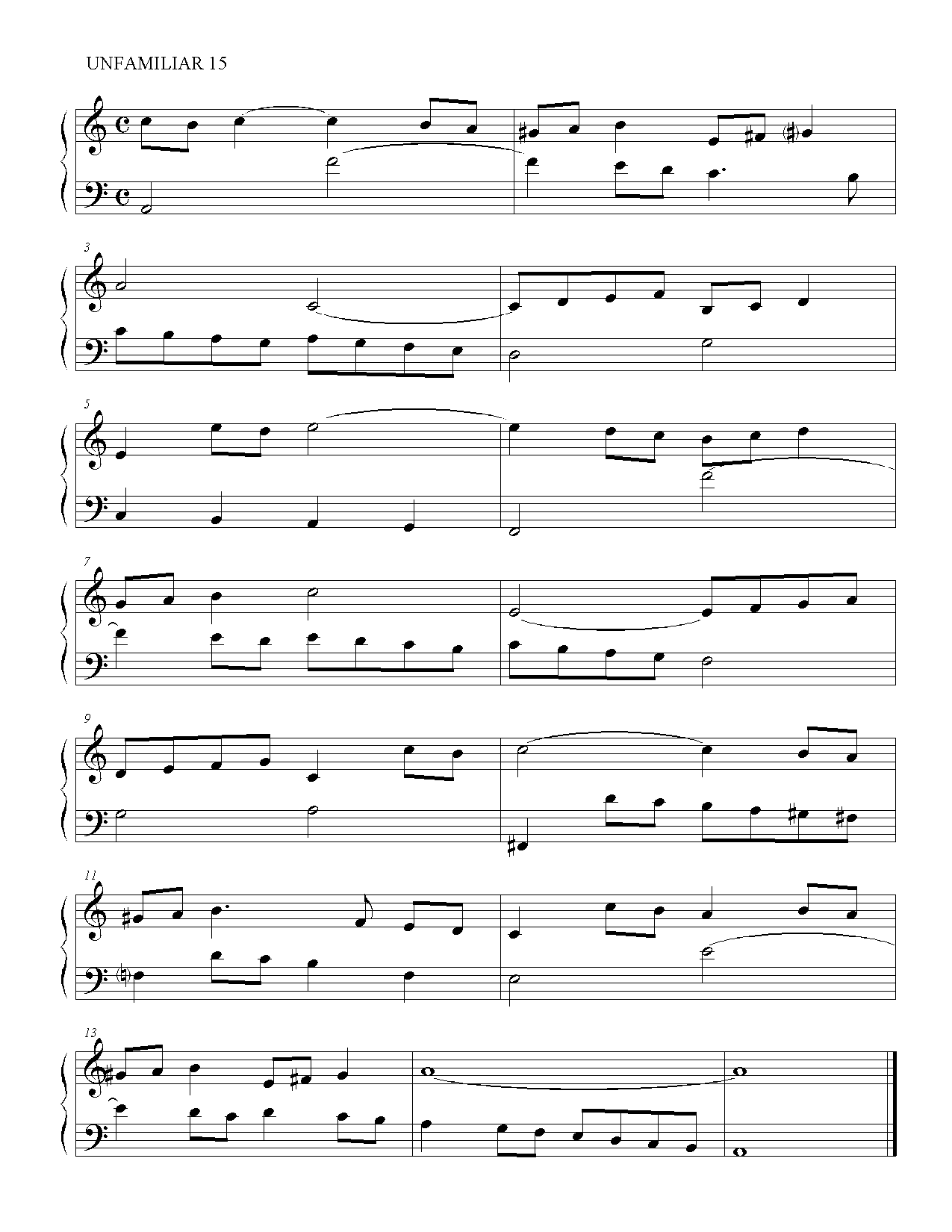

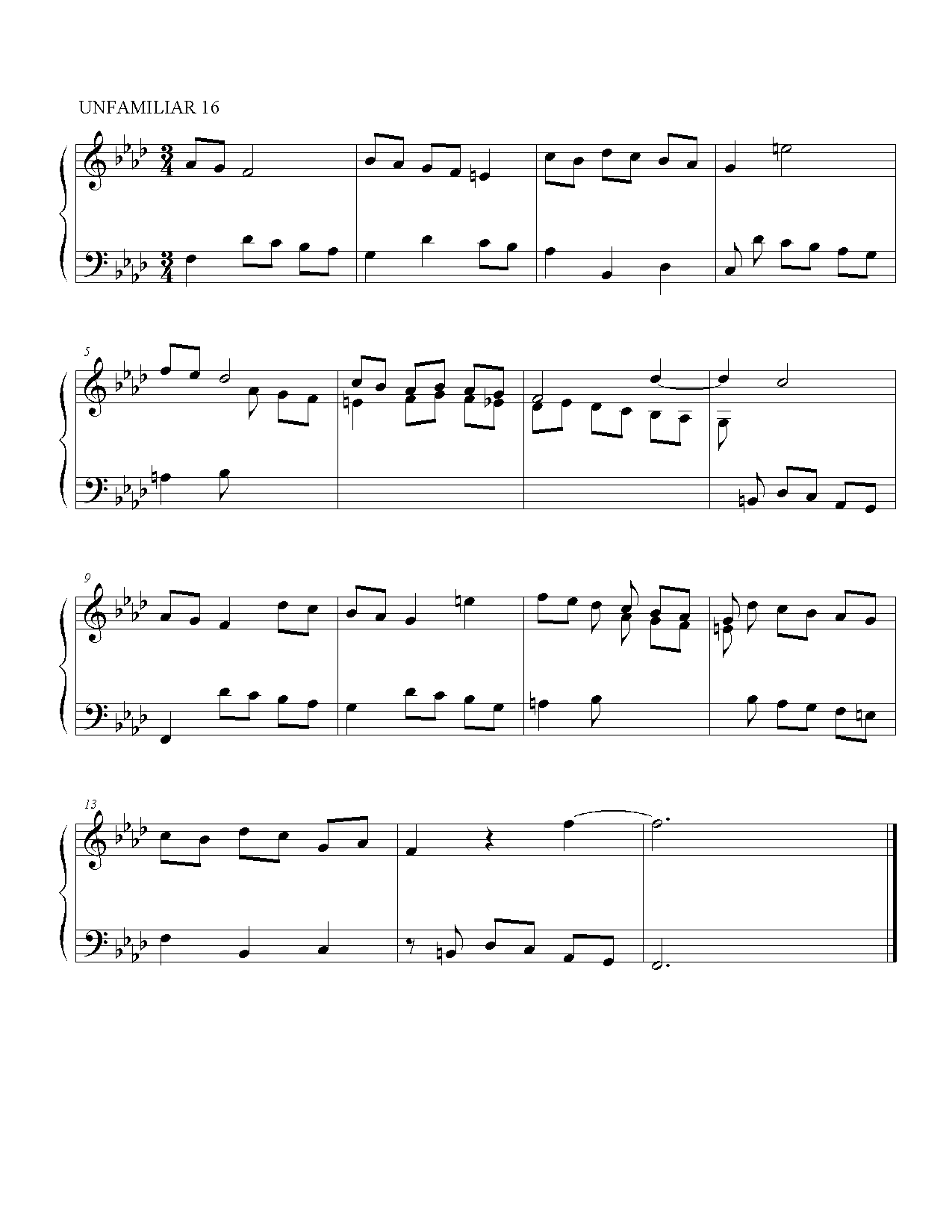

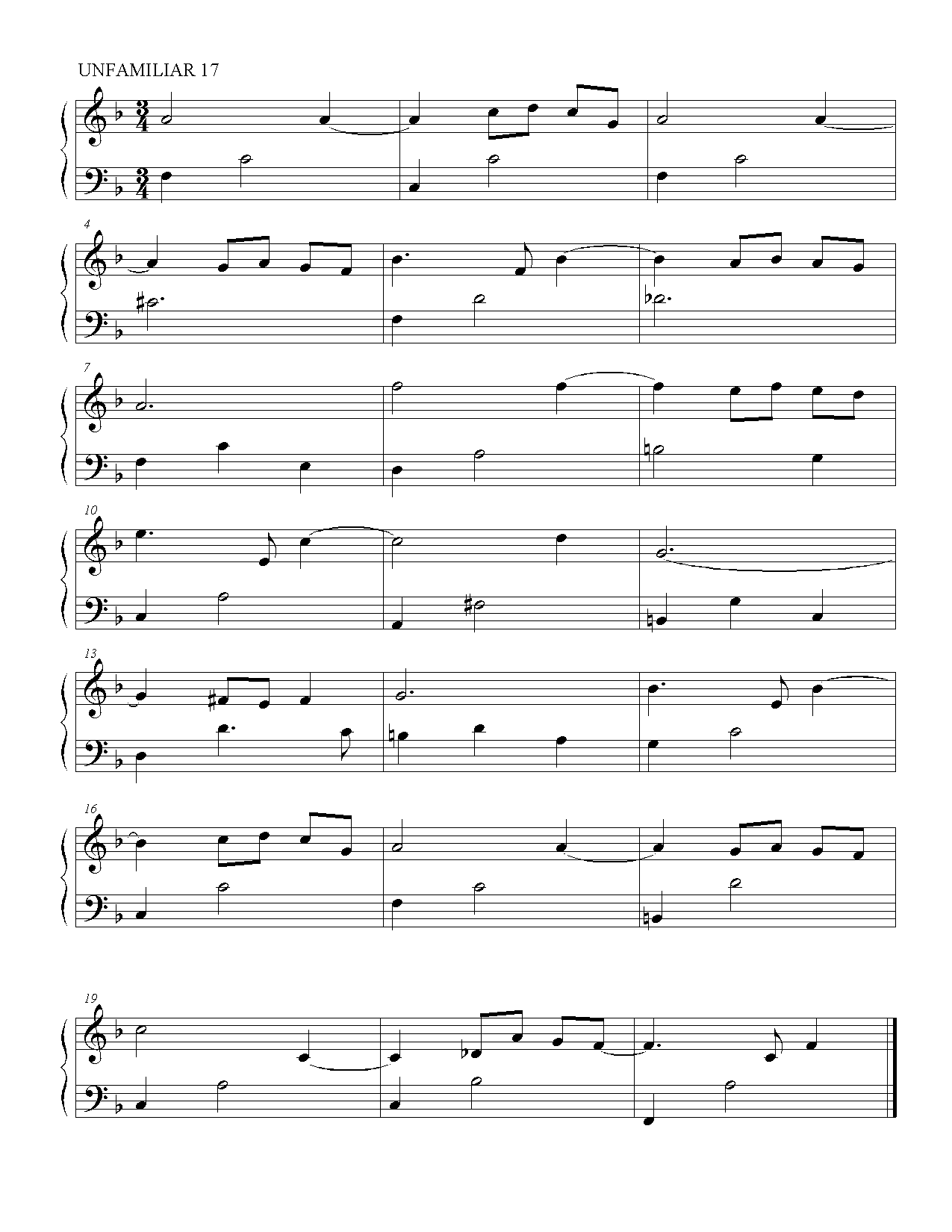

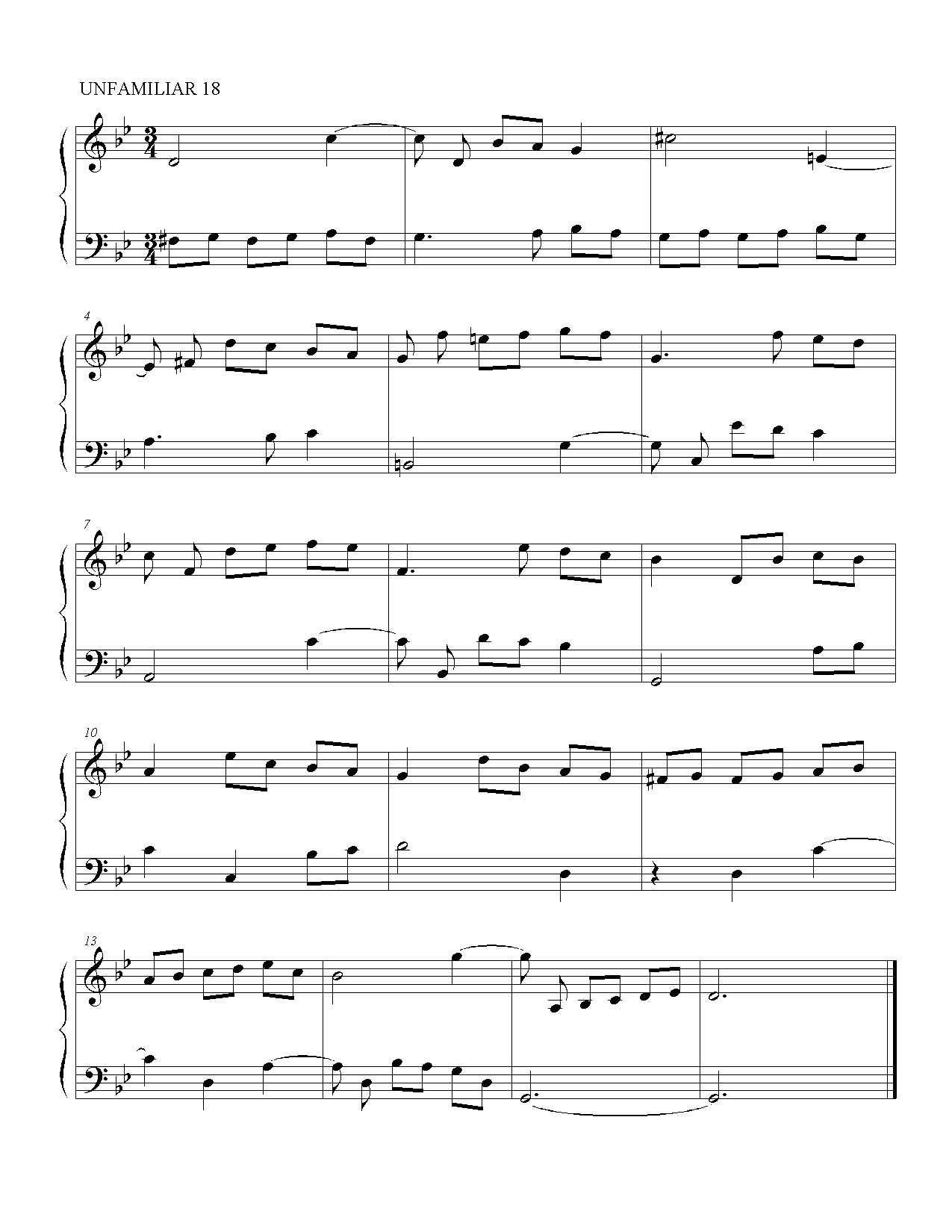

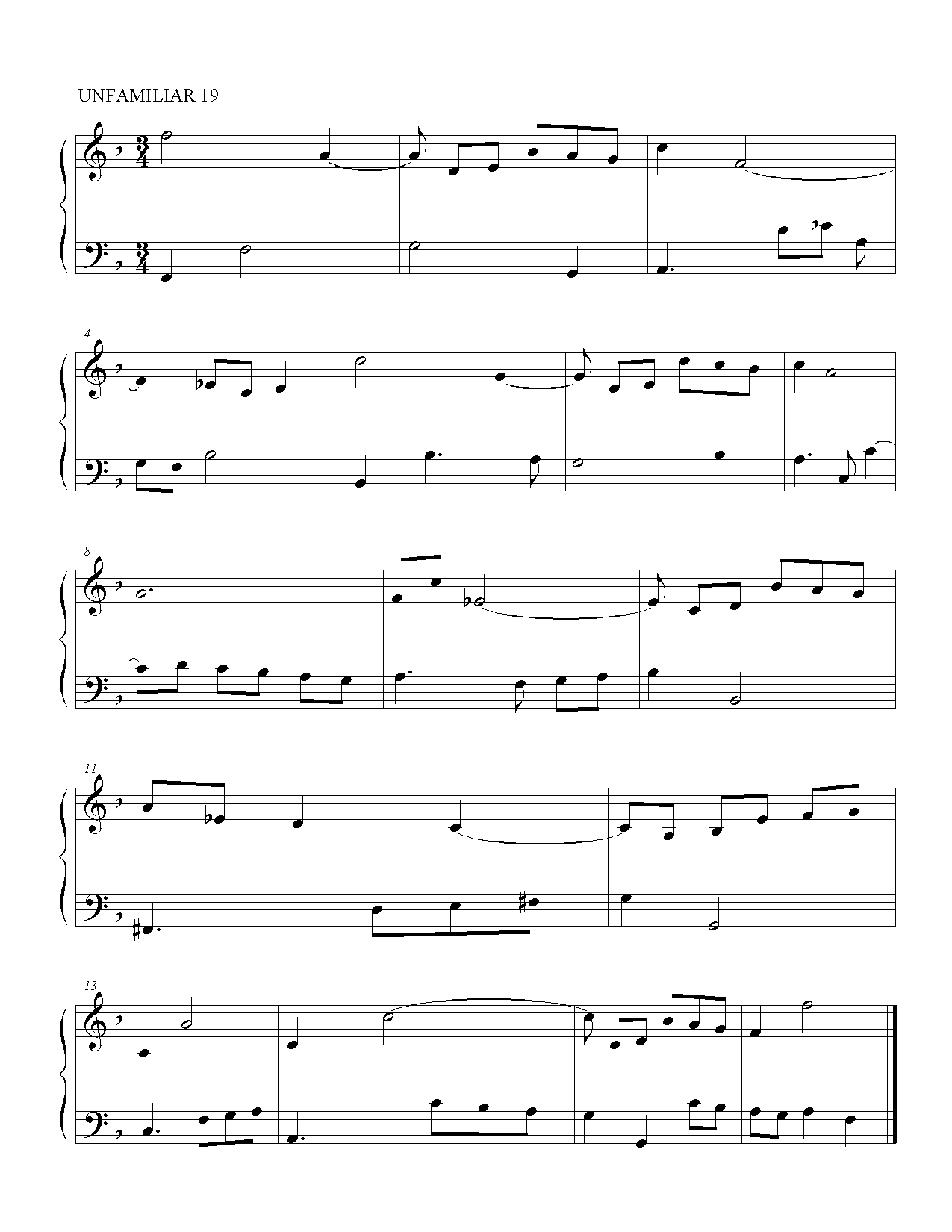

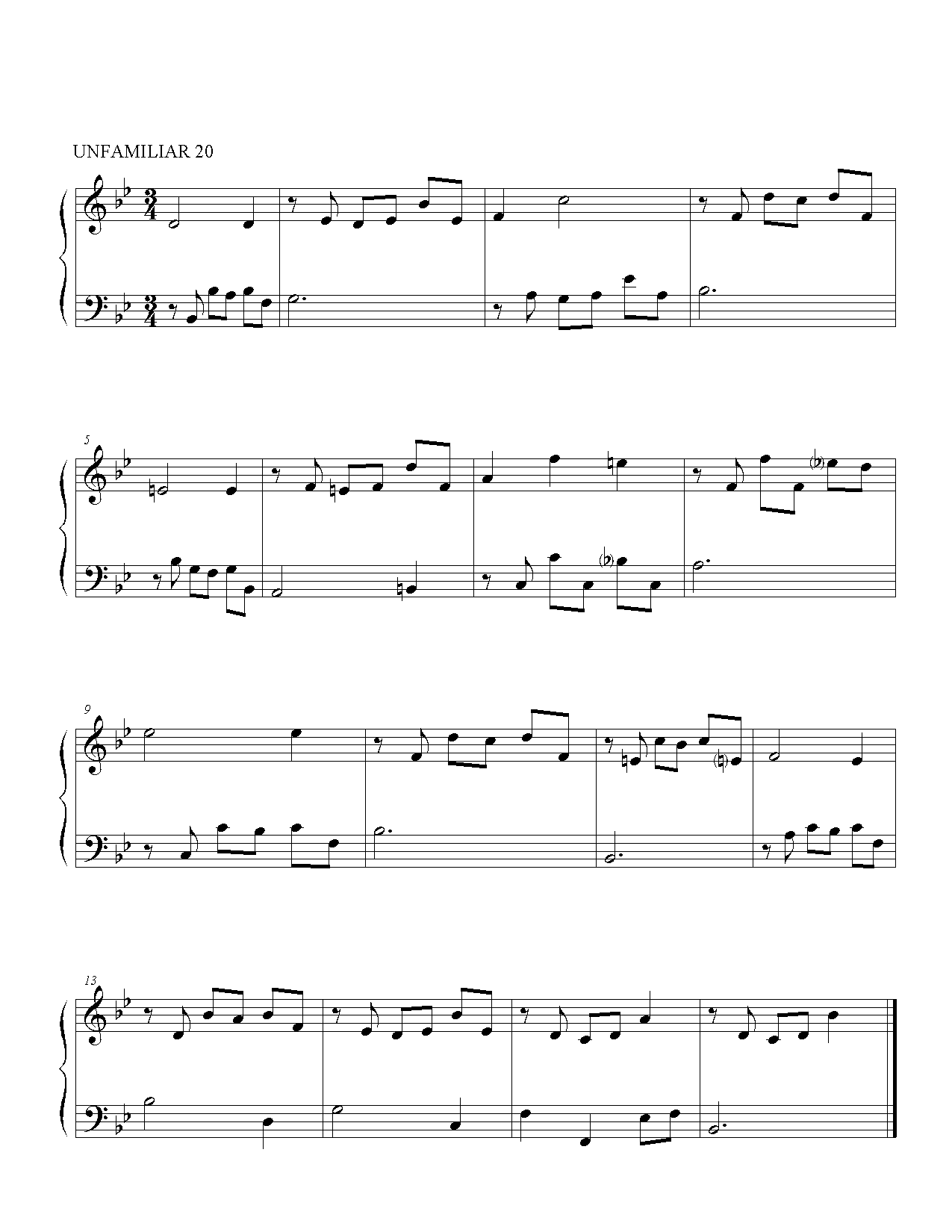

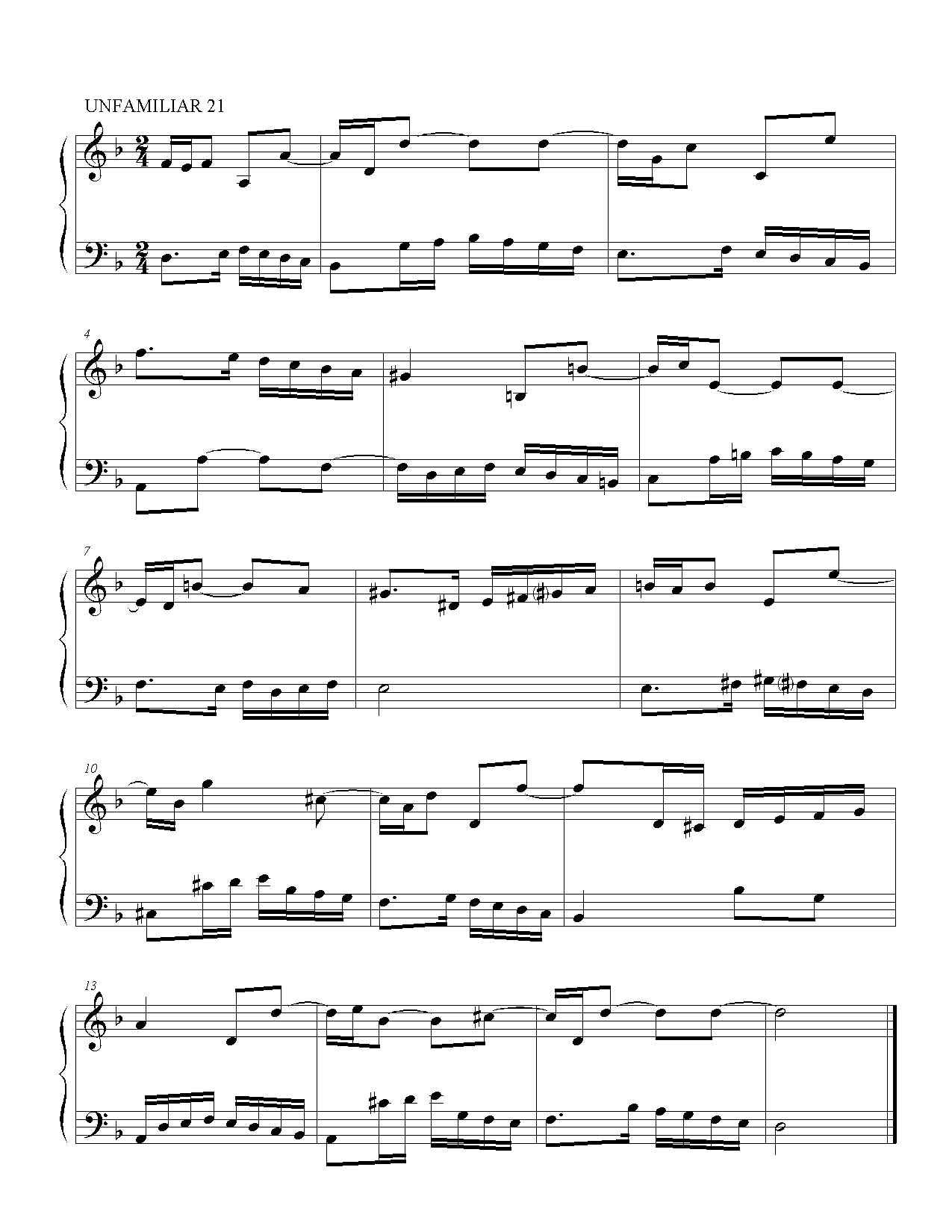

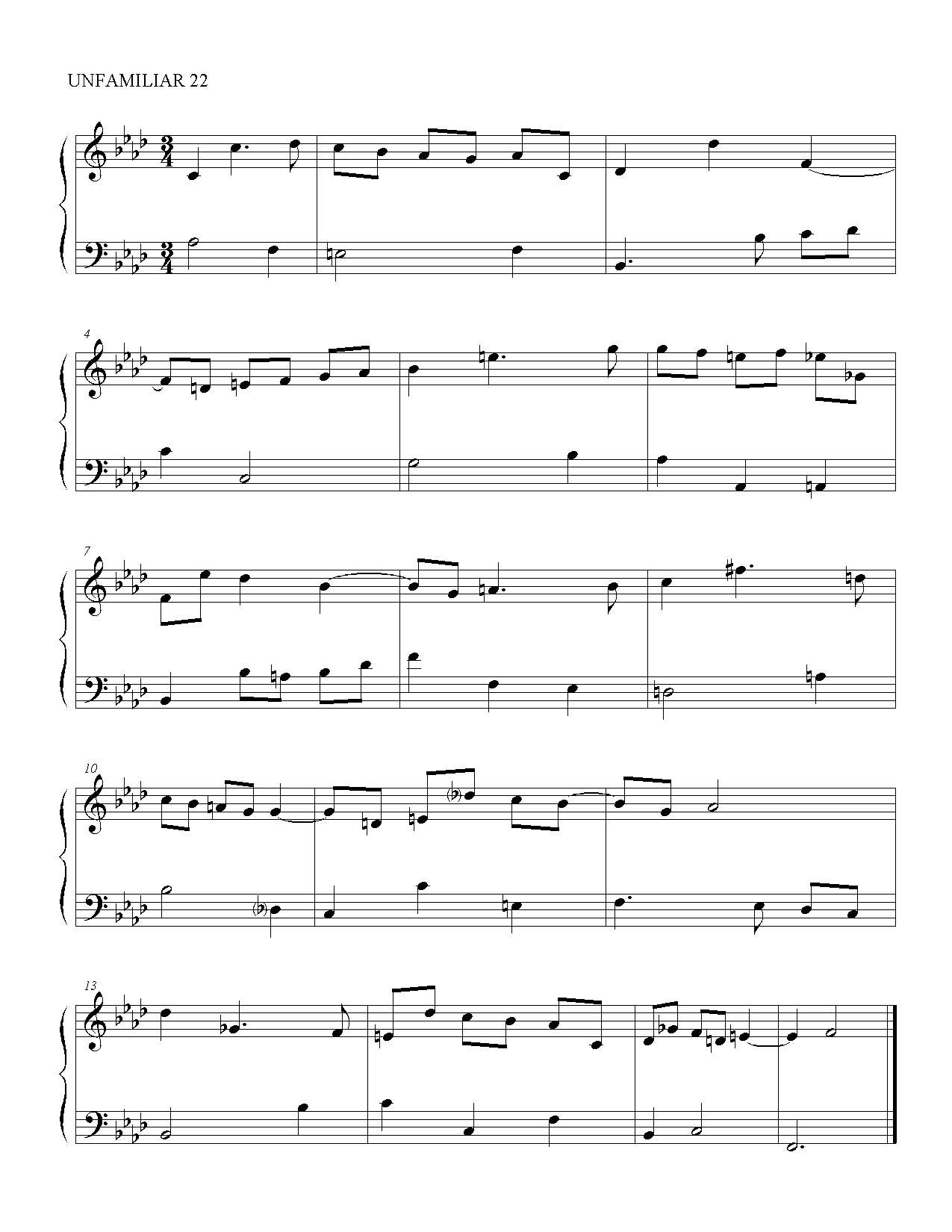

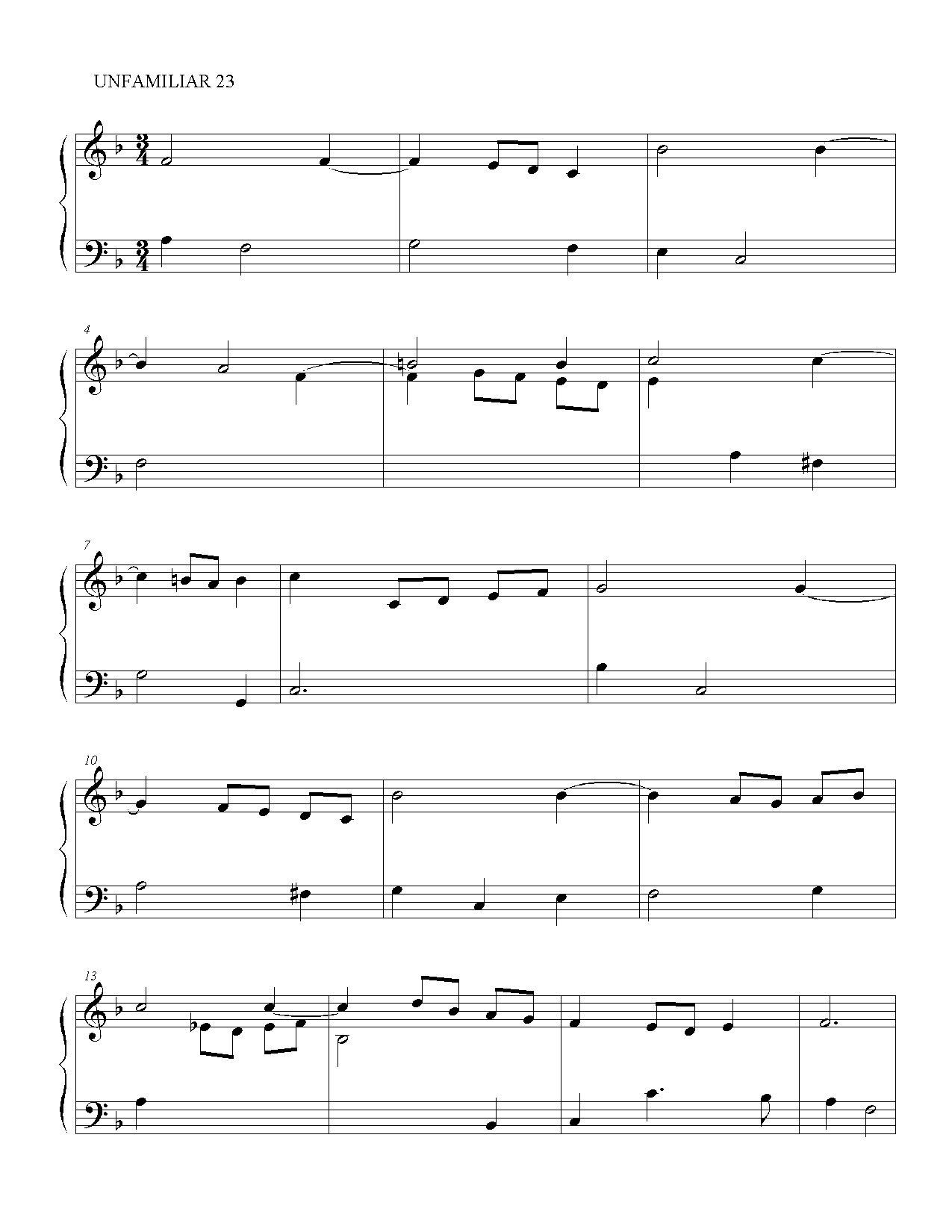

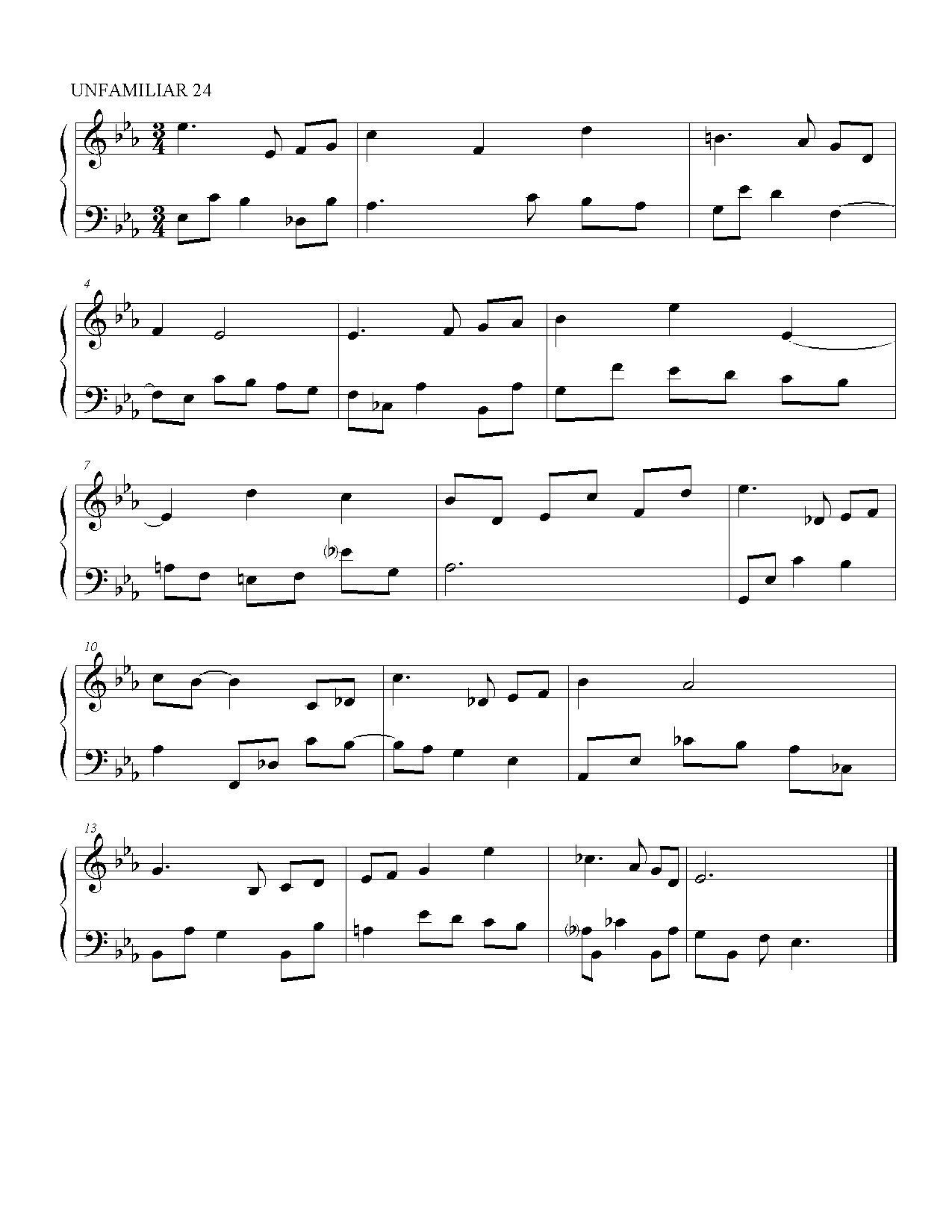

Supplement: Figure S1 — Sores of the new composed (unfamiliar) music excerpts. (DOC) [file pone.0093681.s001.doc]
