# Supplementary material for: Cerebral Activations Related to Audition-Driven Performance Imagery in Professional Musicians
Source: PLoS One. 2014 Apr 8;9(4):e93681. doi: 10.1371/journal.pone.0093681 (PMC3979724; doi:10.1371/journal.pone.0093681)
Supplement: Figure S2 — Scheme of scanning a single trial. (DOC) [file pone.0093681.s002.doc]

**Figure S2. SCHEME OF SCANNING A SINGLE TRIAL.**
