# Supplementary material for: Cerebral Activations Related to Audition-Driven Performance Imagery in Professional Musicians
Source: PLoS One. 2014 Apr 8;9(4):e93681. doi: 10.1371/journal.pone.0093681 (PMC3979724; doi:10.1371/journal.pone.0093681)
Supplement: Table S1 — Titles of familiar music excerpts. (DOC) [file pone.0093681.s003.doc]

**Table S1. TITLES OF FAMILIAR MUSIC EXCERPTS**

| P01 | W.A. Mozart | | | Arietta | KV 24 |
| --- | --- | --- | --- | --- | --- |
| P02 | J.S. Bach | | | Menuet | BWV Anh. 114 |
| P03 | G.F.Händel | | | Vivace | Flute Sonate IX HWV 367b |
| P04 | A. Vivaldi | | | Augelletti, voi col canto... | Cantata Lungi dal vago volto RV68001 |
| P05 | J.S. Bach | | | Gavotte | BWV 808 |
| P06 | W.A. Mozart | | | Menuett | KV 2 |
| P07 | J.S.Bach | | | Bourrée | Lute Sonate BWV 996 |
| P08 | A. Corelli | | | Sarabande | Sonate in D |
| P09 | | G.F. Händel | Contrapunctus | | |
| P10 | J.S.Bach | | | Polonaise | BWV Anh. 119 |
| P11 | J.S.Bach | | | Menuet | BWV Anh. 115 |
| P12 | | I. Bretus | Nocturne | | |
| P13 | | Ch. Du Bois | Arabesque | | |
| P14 | G.F. Händel | | | Bourrée Anglaise | Oboe sonata F major HWV 363a |
| P15 | J.S. Bach | | | Bourrée | BWV 831a |
| P16 | D. Steibelt | | | Leçon Sixième | Méthode de piano (Paris and Leipzig 1809) |
| P17 | J.S. Bach | | | Corrente | BWV 830 |
| P18 | G.F. Händel | | | Minuetto | Oboe sonata F major HWV 363a |
| P19 | G.F. Händel | | | Courante | Suite de Pieces pour le Clavecin |
| P20 | G.F. Händel | | | Allegro | Recorder Sonate Op. 1 Nr. 11 HWV 369 |
| P21 | G.F. Händel | | | Allegro | Violin Sonata HWV 370 |
| P22 | G.F.Händel | | | Presto | Recorder Sonata Nr. 2 HWV 360 |
| P23 | | N. Weiss | Duetto | | |
| P24 | W.Babell | | | Rigaudon | Sonate XII, Solos for a violin or hautboy |

Note that the unfamiliar two-voice excerpts were completely unfamiliar as they were specifically composed for the experiment.
