# Supplementary material for: Cerebral Activations Related to Audition-Driven Performance Imagery in Professional Musicians
Source: PLoS One. 2014 Apr 8;9(4):e93681. doi: 10.1371/journal.pone.0093681 (PMC3979724; doi:10.1371/journal.pone.0093681)
Supplement: Text S1 — Task instructions. (DOC) [file pone.0093681.s004.doc]

**Supporting text S1. TASK INSTRUCTIONS**

- Try not to move during the scan. Make sure you have a comfortable position before the scan starts. After the first twenty minutes, scanning will stop and you will have a chance to find a more comfortable position.

- Keep your eyes closed during the scan. The lights will be off anyway.

- If you want the scan to stop, press the button twice.

- Before each music excerpt is played, you will be told what to do. There are two tasks:

1. Imagine you are playing the piece while you listen to it. Try to imagine you are playing both voices. Don’t sing along or move your fingers. Your hands will be placed on a white cushion, so that the researcher can monitor your possible movements.

2. Give an ongoing commentary on the way the music is being performed. For example, comment on the tuning, synchronization, or interpretation. Verbalize your comments internally. Don’t whisper or speak aloud.

- Between the music excerpts, you will hear a short recording of waves coming in on the beach.

- Every 16 seconds a scan will be made, even while the music is being played. Try to concentrate on the music and not be distracted by the scanner noise.
