# Supplementary material for: Cerebral Activations Related to Audition-Driven Performance Imagery in Professional Musicians
Source: PLoS One. 2014 Apr 8;9(4):e93681. doi: 10.1371/journal.pone.0093681 (PMC3979724; doi:10.1371/journal.pone.0093681)
Supplement: Text S2 — Debriefing after scanning. (DOC) [file pone.0093681.s005.doc]

**Supporting text S2. DEBRIEFING AFTER SCANNING**

The professional musicians reported having been able to ‘play along’ (MoIm) with the pieces bimanually, with the exception of one subject who reported mainly having followed the bass. Two subjects reported difficulty establishing the tonality in the absence of a reference pitch. Subjects reported having no difficulty refraining from finger movements.

Comments on the performances included:

- Visualized the music while playing

- Played slightly after the beat, nevertheless experiencing the hand movements

- Particular difficulty playing along with the unknown pieces

- Didn’t think consciously about the harmony, while playing, but did notice it

- Not aware of having made any movements

- Very concentrated performance, expecially with the unfamiliar pieces

- Scanner noise (in the middle of each piece) troublesome

- Concentration level declined towards the end of the scan

Musicians reported having assessed the presented performance (Judgm) without experiencing any tendency to play along. Subjects verbalized their commentary internally, with the exception of one individual who assessed the performance intuitively without actually verbalizing his comments. One subject described listening ‘as if from a distance.’

Subjects reported assessing:

- Interpretation

- Rhythmic synchronization

- Intonation

- Articulation

- Dynamics

- (Lack of) accents on the first beat

- The wooden quality of the performance

- Ensemble

- Esthetic qualities of the pieces themselves
